# Rhizosphere Microbiomes of European Seagrasses Are Selected by the Plant, But Are Not Species Specific

**DOI:** 10.3389/fmicb.2016.00440

**Published:** 2016-03-31

**Authors:** Catarina Cúcio, Aschwin H. Engelen, Rodrigo Costa, Gerard Muyzer

**Affiliations:** ^1^Microbial Systems Ecology, Department of Aquatic Microbiology, Institute for Biodiversity and Ecosystem Dynamics, University of AmsterdamAmsterdam, Netherlands; ^2^Marine Ecology and Evolution Research Group, Centro de Ciencias do Mar, Universidade do AlgarveFaro, Portugal; ^3^Microbial Ecology and Evolution Research Group, Centro de Ciencias do Mar, Universidade do AlgarveFaro, Portugal

**Keywords:** 16S rRNA, amplicon sequencing, marine bacteria, rhizosphere, seagrass microbiome, sulfur bacteria, sulfur cycle, plant–microbe interactions

## Abstract

Seagrasses are marine flowering plants growing in soft-body sediments of intertidal and shallow sub-tidal zones. They play an important role in coastal ecosystems by stabilizing sediments, providing food and shelter for animals, and recycling nutrients. Like other plants, seagrasses live intimately with both beneficial and unfavorable microorganisms. Although much is known about the microbiomes of terrestrial plants, little is known about the microbiomes of seagrasses. Here we present the results of a detailed study on the rhizosphere microbiome of seagrass species across the North-eastern Atlantic Ocean: *Zostera marina, Zostera noltii*, and *Cymodocea nodosa.* High-resolution amplicon sequencing of 16S rRNA genes showed that the rhizobiomes were significantly different from the bacterial communities of surrounding bulk sediment and seawater. Although we found no significant differences between the rhizobiomes of different seagrass species within the same region, those of seagrasses in different geographical locations differed strongly. These results strongly suggest that the seagrass rhizobiomes are shaped by plant metabolism, but not coevolved with their host. The core rhizobiome of seagrasses includes mostly bacteria involved in the sulfur cycle, thereby highlighting the importance of sulfur-related processes in seagrass ecosystems.

## Introduction

In recent years many studies have been conducted on the composition, activity, and interaction of bacterial communities and terrestrial plants (e.g., Egamberdieva et al., 2008; Bulgarelli et al., 2012; Philippot et al., 2013; Chaparro et al., 2014; Ofek-Lalzar et al., 2014). Most of this research has been focused on the rhizosphere, a thin zone of soil under direct influence of root exudates, also known as ‘rhizodeposits’ (Martens, 1990). The type and quantity of rhizodeposits shape the composition of bacteria present in the rhizosphere, the ‘rhizobacteria’ (Kloepper and Schroth, 1979; Lugtenberg and Kamilova, 2009). These rhizobacteria are a diverse mixture of microorganisms that can actively interact with the plant in different ways and include both pathogenic and pathogen-suppressing bacteria, as well as bacteria that can enhance the plant’s fitness for example by releasing growth-promoting factors (Mendes et al., 2011, 2013; Berendsen et al., 2012; Bulgarelli et al., 2013) or by fixing nitrogen (Spaink, 2000; Mehnaz et al., 2007; Miller et al., 2007).

Although the rhizosphere microbiome of terrestrial plants is well studied, little is known about the bacteria living in close association with marine plants, such as seagrasses. Seagrasses are a paraphyletic group of angiosperms, which recolonized the marine environment. Their primary and secondary metabolism is similar to that of terrestrial plants due to their common evolutionary origin (Agostini et al., 1998; Heglmeier and Zidorn, 2010). Four families within the order *Alismatales*, the *Cymodoceaceae*, *Hydrocharitaceae*, *Posidoniaceae*, and *Zosteraceae*, exclusively contain marine plant species (den Hartog and Kuo, 2006), which are distributed mostly in soft-bottom sediments of intertidal and shallow sub-tidal areas from tropical to cold-temperate coastal zones (Fonseca et al., 2000; Papenbrock, 2012). Seagrasses are important ecosystem engineers that provide feeding grounds and habitats to a large variety of marine organisms. Their canopies and dense meadows are responsible for trapping substantial amounts of sediment particles and organic matter (Gacia et al., 1999) enhancing water clarity, and account for roughly 10% of the yearly global carbon sequestration in marine sediments (Duarte et al., 2005b; Kennedy et al., 2010; Fourqurean et al., 2012).

Sediments inhabited by seagrasses are typically anoxic and highly reduced due to the presence of sulfide, which is a strong phytotoxin (Koch and Erskine, 2001) responsible for die-off events of seagrasses (Borum et al., 2005; Krause-Jensen et al., 2011). Sulfide is a product from the activity of sulfate-reducing bacteria that use sulfate as a terminal electron acceptor for the mineralization of organic matter (Capone and Kiene, 1988). The availability of diverse terminal electron acceptors and the exudates released by seagrass roots stimulate bacterial growth and promote a series of microbial-mediated redox processes in the rhizosphere, resulting in an extensive range of microenvironments readily available for the establishment of complex microbial communities (Isaksen and Finster, 1996; Holmer and Nielsen, 1997; Blaabjerg and Finster, 1998; Hansen et al., 2000; Devereux, 2005; Duarte et al., 2005a).

Some studies have investigated the belowground microbial diversity associated with seagrass meadows including rhizosphere, rhizoplane, and endosphere (e.g., Cifuentes et al., 2000, 2003; James et al., 2006; Jensen et al., 2007; Crump and Koch, 2008; Mejia et al., 2016), and these belowground niches have been described to be dominated mostly by members of the classes *Alpha*-, *Gamma-*, *Delta-*, *Epsilonproteobacteria*, and/or *Bacteroidetes* (Cifuentes et al., 2000; Jensen et al., 2007; Mejia et al., 2016). Nonetheless, the majority of these studies have been performed on a small number of seagrass species, and it is not always clear which structure was sampled.

Here, we used high-resolution amplicon sequencing of 16S rRNA genes to characterize, for the first time, the rhizosphere microbiome (hereinafter referred to as rhizobiome) of seagrasses from the North-eastern Atlantic Ocean: the common eelgrass *Zostera marina*, the dwarf eelgrass *Zostera noltii*, and the little Neptune grass *Cymodocea nodosa.* We studied the variation between rhizobiomes of *Z. marina* and *Z. noltii* from the South of Portugal and the North of France, and characterized the core rhizobiome of North-eastern Atlantic seagrasses. In addition, we discussed the possible niche occupation of sulfur bacteria in these rhizobiomes, due to the importance of the sulfur cycle on the fitness and survival of seagrasses.

## Materials and Methods

### Description of Sampling Sites

Rhizospheres from *Zostera marina* (Zm/ZmPt), *Z. noltii* (Zn/ZnPt) and *Cymodocea nodosa* (Cn/CnPt), as well as bulk sediment (Sed/SedPt) and seawater (SW/SwPt) were sampled from the intertidal regions of Culatra Island (Faro, Portugal, 36°59′56.0″ N 7°49′31.7″ W) in July 2013. The three seagrass species collected in Portugal were located in adjacent meadows; therefore bulk sediment and seawater samples were obtained from one single site on their surroundings. Culatra Island is one of five islands in the Ria Formosa lagoon, a meso-tidal system with a surface area of 84 km^2^ (Cabaço and Santos, 2010). This island is one of the few locations in the Ria Formosa where these three seagrasses coexist. They inhabit mostly sandy sediments (Asmus et al., 2000), although there is a high variability in sediment characteristics at a very small scale (Falcão and Vale, 1990). The salinity is typically 36 PSU although it can sporadically decrease to 27 PSU, and seawater temperature ranges between 12°C in winter and 27°C in summer (Newton and Mudge, 2003).

In order to compare rhizobiomes across regions, rhizospheres of *Z. marina* were collected from Pointe de Cléguer (Zm/ZmFr, Roscoff, France, 48°43′37.1″ N 3°58′35.9″ W) and *Z. noltii* from Penar Vil (Zn/ZnFr, Roscoff, France, 48°41′27.4″ N 3°57′24.9″ W), in September 2013. Two sets of bulk sediments were collected, one in the surroundings of *Z. marina* (SedM, SedMFr) and another one in the surroundings of the *Z. noltii* meadow (SedN, SedNFr). *Cymodocea nodosa* was not sampled from Roscoff, due to its Mediterranean-Atlantic geographical distribution. Roscoff, which is located in northern Brittany, France, has a mega-tidal system with a patchy coverage of *Z. noltii* and *Z. marina* beds growing in sandy sediments (median grain size: 252 ± 10 and 302 ± 11 μm, respectively; Ouisse et al., 2010) with low organic matter content (1.12 ± 0.19 and 1.86 ± 0.51 %, respectively; Ouisse et al., 2012). Salinity varies between 21 and 35 PSU, and seawater temperature between 7 and 23°C (Bachelet et al., 1992; Li et al., 2009).

### Sampling Strategy

Five 15 cm diameter cores of each seagrass species were randomly collected during low tide. In addition, bulk sediments were collected from a depth of 1–11 cm representing the root zones of the seagrasses, using 50 mL syringes (*n* = 5), and seawater (1 L, *n* = 5). To obtain the rhizosphere from the seagrasses we adapted a method that is commonly used for the retrieval of rhizospheres from terrestrial plants (e.g., Shieh and Yang, 1997; Costa et al., 2006; Lundberg et al., 2012). Briefly, each seagrass core was slowly emptied in a tray, maintaining the structure of the sectioned sediment intact.

Sandy sediments, such as those present in the sampling areas (Ouisse et al., 2012), are strongly trapped within the root structure of the seagrasses, in particular by *Z. noltii*. Therefore, the complex network of roots was carefully separated by gently shaking the tray sideways, and 4–6 shoots from each core were selected for rhizosphere recovery. Thereafter, roots from the selected seagrasses were manually shaken in order to remove loose sediment (which was excluded from the collection), and the sediment that remained attached to the roots (rhizosphere) was collected for further analysis by washing the roots with 0.2 μm-filtered seawater. Additionally, 5 cm diameter cores were collected in triplicate from each seagrass and bulk sediment, for sediment characterization. Samples were transported to the laboratory in a cool-box and immediately processed on arrival.

### DNA Extraction and 16S Amplicon Sequencing

For each of the five seawater replicates, 600 mL of seawater was filtered through a 0.2 μm-pore size nitrocellulose filter to collect microbial biomass. Rhizosphere and bulk sediment samples were checked for meiofauna and plant detritus, which were removed if present. Thereafter they were processed in a Stomacher 80 Laboratory Blender (Seward Medical) for 3 cycles of 1 min at normal speed, in order to release microbial cells from the mineral particles (Buesing and Gessner, 2002). The supernatant was centrifuged for 30 min at high speed (10,000 *g*) (Costa et al., 2006), after which the pellet containing the microbial biomass was processed immediately. The DNA from filters and microbial pellets from rhizosphere and bulk sediments was extracted using the PowerSoil DNA Isolation Kit (MO BIO Laboratories, Inc., Carlsbad, CA, USA), following the manufacturer’s instructions. The isolated DNA was quantified using a dsDNA HS Assay Kit on a Qubit 2.0 Fluorometer (Life Technologies, Grand Island, NY, USA).

Sequencing was performed on an Illumina MiSeq system by the company Research and Testing Laboratory (Lubbock, TX, USA). The 16S rRNA gene libraries were prepared according to a modified protocol from Kozich et al. (2013). Briefly, amplification was performed using a forward and reverse fusion primer. The primer pair S-D-Bact-0341-b-S-17, 5′-CCTACGGGNGGCWGCAG-3′ and S-D-Bact-0785-a-A-21, 5′-GACTACHVGGGTATCTAATCC-3′ (Herlemann et al., 2011) was used to generate 464 bp paired-end reads, covering the V3-V4 16S rRNA region. The forward primer S-D-Bact-0341-b-S-17 was complemented with an Illumina i5 adapter (5′- AATGATACGGCGACCACCGAGATCTACAC-3′), and the reverse primer with an i7 adapter (5′-CAAGCAGAAGACGGCATACGAGAT-3′). Furthermore, an 8–10 bp barcode and a primer pad sequence were added to each primer. The pad sequences were designed in order to guarantee a melting temperature between 63 and 66°C for each primer/pad combination. Amplifications were performed using the Qiagen HotStar Taq master mix (Qiagen Inc, Valencia, CA, USA), 1 μl of each primer (5 μM) and 1 μl of template, in a total volume of 25 μl per reaction. PCR amplification started with an initial denaturation step at 95°C for 5 min, followed by 35 cycles at 94°C for 30 s, 54°C for 40 s and 72°C for 1 min. The reaction was stopped after a final extension step at 72°C for 10 min. Subsequently, the amplicons were visualized on an eGel, pooled and quantified before they were loaded on an Illumina MiSeq system (Illumina, Inc. San Diego, CA, USA). A total of 2,566,312 reads were generated from the 45 samples, with an average sequence length of 427 bp. After quality filtering, a total of 1,065,217 sequences remained, with an overall average of 23,671 ± 7,561 sequences per sample and 6,950 ± 1,856 unique sequences per sample.

### Post-sequencing Analysis

Post-sequencing analyses were performed on each biological replicate (Prosser, 2010). Sequencing data was analyzed using QIIME (version 1.7.0, Caporaso et al., 2010b). Briefly, raw sequences with merged paired-ends were demultiplexed and quality filtered. Primer detection was disabled in QIIME, because MiSeq sequencing does not sequence the primers. Subsequently, the sequences were clustered in operational taxonomic units (OTUs) using UCLUST (Edgar, 2010) with a sequence similarity threshold of 97%. A representative set of OTUs was selected and aligned using the PyNAST algorithm (Caporaso et al., 2010a). All sequences that failed the alignment and singleton OTUs were not included in the analyses. Taxonomy assignment was performed with RDP classifier using the May 2013 release of Greengenes as a reference dataset (http://greengenes.lbl.gov/). An OTU table was built excluding sequences with no hit or matching chloroplast DNA. Core OTUs were defined as those present in 100% of the seagrass samples, including all 5 replicates of each species/location.

The sequencing method and data analysis applied in this work resulted in high quality reads; however we acknowledge the biases associated with amplicon-based sequencing methods, and the presence of multiple sequence copies. Nevertheless, the results here described were identical and comparable to those obtained through the analysis of 16S rDNA reads obtained by Illumina shotgun sequencing of pooled replicates from four of the samples here analyzed. Moreover, the quality check and singleton removal abovementioned enabled us to provide a reproducible and reliable description (quantitatively and qualitatively) of the seagrass rhizobiome. Alpha diversity measures (Shannon Index of diversity and observed OTUs) were calculated based on rarefied OTUs, with 8055 sequences per sample, the maximum number of sequences common to all samples.

Differences between samples (beta diversity) were calculated in STAMP v2.0.8 (Parks et al., 2014), using the data generated by QIIME’s taxonomy assignment and corresponding mapping file. STAMP calculated diversity differences between samples based on the analysis of variances (ANOVA) with a significant level of *P* < 0.05, and significant differences between groups of samples were investigated using Scheffé’s *post hoc* test. Benjamini–Hochberg’s False Discovery Rate (FDR) was applied to correct for multiple comparisons.

Permutational Multivariate Analysis of Variance (PERMANOVA) was applied to identify compositional differences between regions and seagrass species with a significance level of *P* < 0.01, based on a Bray–Curtis distance matrix calculated in PAST software version 2.17c (Hammer et al., 2001). Similarity Percentage method (SIMPER, Clarke, 1993) was used to assess which taxa were responsible for differences observed between two groups of samples (pairwise SIMPER) or between all the samples pooled (multi-group SIMPER), using a Bray-Curtis dissimilarity matrix, calculated, as abovementioned, in PAST software.

The amplicon sequences have been deposited as dataset SRP057630 in the Sequence Read Archive (SRA, EMBL).

### Abiotic Characterization of Sediments from Portugal

Grain size determination was performed by standard sieve fractionation of the top 5 cm of small sediment cores, as described by Holme and McIntyre (1984). Due to the agglomeration of very fine particles (<43 μm) and blockage of the sieve during dry sieving, wet sieving was manually performed on pre-weighed, oven-dried sediments (105°C overnight), through a 63 μm mesh size (Krause-Jensen et al., 2011). Wet-sieved sediments were again dried at 105°C and sieved in a throw- action sieve shaker (10 min, amplitude 30 mm; AS Basic, Retsch GmbH Haan), through 6 different mesh sieves (0.063, 0.125, 0.25 0.5, 1, and 2 mm). Each size fraction was weighed, and the curve of cumulative percentages of each fraction was used to calculate median grain size. Sediment classification was subsequently performed according to Wentworth’s grain size classes (Wentworth, 1922).

Water content was calculated after drying the top 1 cm of sediment at 105°C overnight, and loss on ignition (LOI) followed by overnight combustion at 375°C (Sutherland, 1998). The majority of studies determine LOI through combustion at 520 or 550°C, however it has been reported that loss of structural water from metal oxides and clay minerals can take place at temperatures as low as 400°C (Dean, 1974; Sutherland, 1998). Due to a high percentage of small particles in some of the samples (<63 μm), we considered that combustion at 375°C would provide more accurate results.

Differences between water content, LOI and grain size of sediments from Portugal, as well as grain size from France were analyzed using a one-way ANOVA, and differences between pairs of samples were assessed with Tukey’s HSD *post hoc* test with a significance level of *P* < 0.05. In order to investigate differences in organic content between Portugal and France, a two-sample *t*-test was performed on the online tool http://in-silico.net.

## Results

### Sediment Characteristics

**FIGURE 1:**
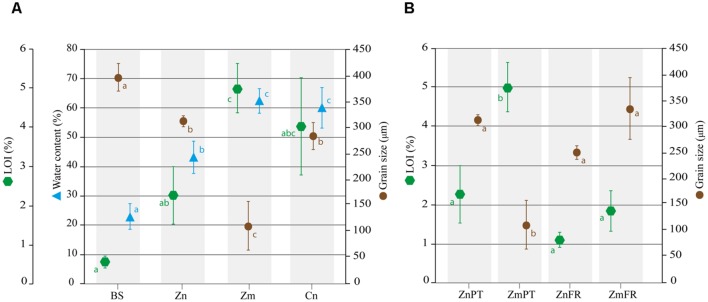
**Abiotic characterization of sediments from the study areas in Portugal and France.** Mean percentages of water content, loss on ignition (LOI), as well as median grain size are shown for bulk sediments (BS) and sediments of *Z. noltii* (Zn), *Z. marina* (Zm), and *C. nodosa* (Cn) sampled in Portugal **(A)**. Comparison of LOI and grain size *Zostera* spp. sediments between Portugal and France. The data of ZnFr and ZmFr were obtained from Ouisse et al. (2010, 2012) **(B)**. Bars indicate standard deviations, and letters reveal presence/absence of significant relationships between samples on each analysis with a significance level of 0.05, as determined by Tukey’s HSD *post hoc* test.

Standard sieve fractionation revealed that the grain size of sediments sampled in Portugal significantly differed between distinct sources of samples (ANOVA, *P* = 0.0002e-01). Based on Wentworth’s size classes, sediments of *Z. noltii* (mean ± SD, 311 ± 11 μm), *C. nodosa* (284 ± 26 μm) and bulk sediments (396 ± 26 μm) were classified as medium sand, whereas sediment of *Z. marina* was classified as very fine sand (111 ± 47 μm). Nevertheless, significant sediment composition differences were detected among all samples except between *Z. noltii* and *C. nodosa* (*P* = 0.6704, **Figure 1A**). Regarding grain size distribution of *Zostera* sp. sediments in Portugal and France, the only significant difference detected was between *Z. marina* sampled in Portugal, and all other samples (*P* < 0.001, **Figure 1B**).

Water content was significantly lower in bulk sediments and *Z. noltii* than in *Z. marina* and *C. nodosa* (*P <* 0.01), and did not differ between the latter two species (*P* = 0.9000, **Figure 1A**).

The organic content inferred from LOI at 375°C was significantly higher in sediments of *Z. marina* and *C. nodosa* than in bulk sediments (*P* = 0.0010 and *P* = 0.0031, respectively, **Figure 1A**), however, pairwise comparison of the latter with sediments from *Z. noltii* revealed no significant differences between them (*P* = 0.1148, **Figure 1A**). Following the same trend observed in the comparison of grain size distribution between Portugal and France, only *Z. marina* sediments from the former location were significantly different from all other samples (*P* = 0.0032, **Figure 1B**).

### Microbial Community Composition of Rhizospheres and Surrounding Environment

**FIGURE 2:**
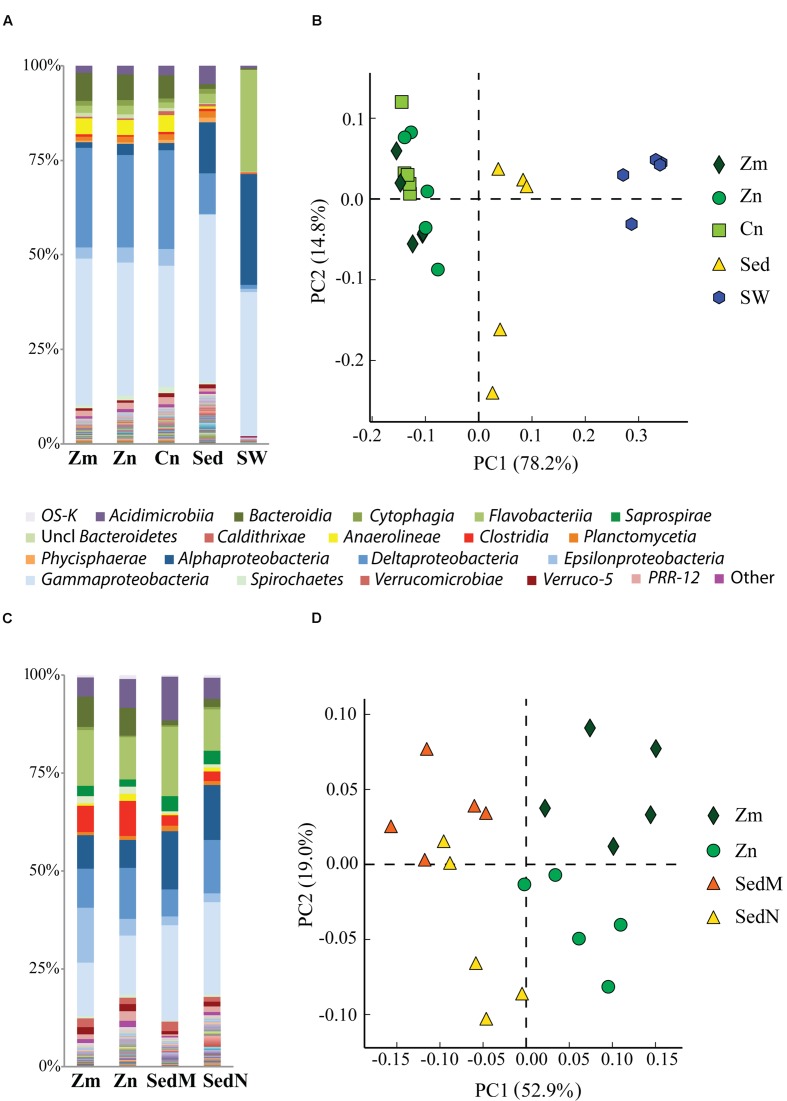
**Comparison of bacterial communities of the seagrass rhizosphere (*Z. marina*, Zm; *Z. noltii*, ZN; *C. nodosa*, CN) and the surrounding environment (bulk sediment, Sed; and seawater (SW) from Portugal and France.** Bulk sediments from the surroundings of *Z. marina* and *Z. noltii* are distinguished (SedM and SedN, respectively). Shown are the bacterial community compositions (average of five independent samples) from **(A)** Portugal and **(C)** France, and principal component analysis (PCA) of the bacterial communities from **(B)** Portugal and **(D)** France. Both PCAs show a clear separation between the bacterial communities of the rhizosphere and those from the surrounding environment. Only classes represented by an average abundance above 0.5 % are shown on the legend of the bar graphs **(A,C)**, and the percentage of community variance explained by each axis is indicated in parentheses **(B,D)**.

**FIGURE 3:**
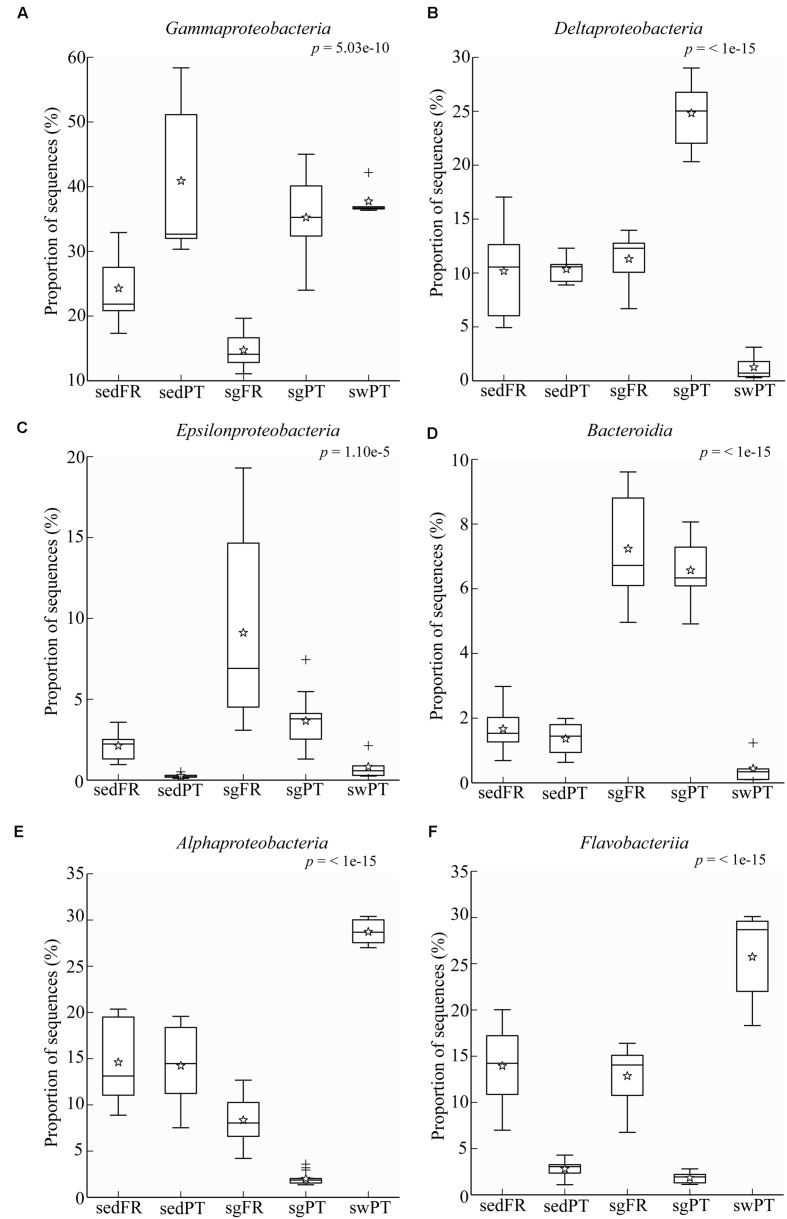
**Box plots showing the relative abundance of sequences of the most abundant groups in the rhizobiome of seagrasses from Portugal and France, and the bacterial communities from the surrounding environment.** Indicated are bulk sediments from Portugal (sedPT) and France (sedFR); seagrasses from Portugal (sgPT) and France (sgFR); seawater from Portugal (swPT). The top of the box indicates the third quartile, the bottom the first quartile, and the line in the middle is the median. The star indicates the mean of the data, crosses are outliers, and the whiskers represent error bars. **(A)** *Gammaproteobacteria*, **(B)** *Deltaproteobacteria*, **(C)** *Epsilonproteobacteria*, **(D)** *Bacteroidia*, **(E)** *Alphaproteobacteria*, and **(F)** *Flavobacteriia*.

Comparative analysis of the amplicon sequences showed in both regions strong differentiation in bacterial communities between the seagrass rhizobiomes and those present in bulk sediments and seawater (Portugal: PERMANOVA, *P* = 0.0006; **Figures 2A,B**; France: PERMANOVA, *P* = 0.0001; **Figures 2C,D**). The rhizobiomes were dominated by members of the classes *Gamma-* (**Figure 3A**), *Delta-* (especially in Portugal; **Figure 3B**), and *Epsilonproteobacteria* (especially in France; **Figure 3C**), and *Bacteroidia* (**Figure 3D**), while the seawater was dominated by members of the *Alpha*- (**Figure 3E**), *Gammaproteobacteria* and *Flavobacteriia* (**Figure 3F**). Communities from the bulk sediments were predominantly composed of members from the *Alpha-*, *Gamma-*, and *Deltaproteobacteria* classes.

Rarefaction analysis showed that seawater accounted for the lowest diversity (Shannon index = 7.1, and 1803 observed OTUs on rarefied samples). Furthermore, in general, the diversity of bulk sediments did not differ from the rhizosphere (Shannon index of 10.33 and 10.32, respectively; PERMANOVA, *P* = 0.8749). On the other hand, although not statistically significant, the OTU richness was higher in bulk sediments than in rhizospheres (3522.5 and 3471.5 observed OTUs, respectively; PERMANOVA, *P* = 0.6944).

### Rhizobiomes of Different Seagrasses Within and Across Regions

**FIGURE 4:**
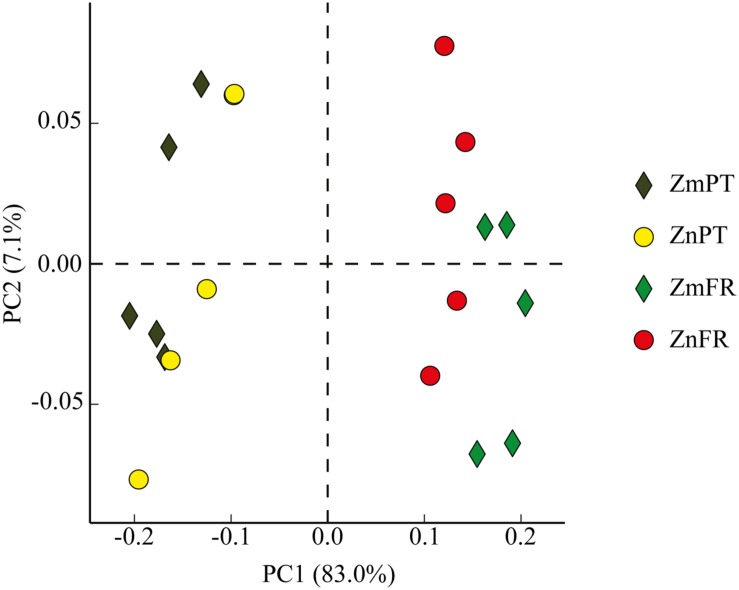
**Principal component analysis (PCA) plot comparing bacterial communities of the rhizosphere of the seagrasses *Z. marina* (Zm) and *Z. noltii* (Zn), from Portugal (PT) and France (FR).** Percentage of community variance explained by each axis is indicated in parentheses.

Principal component analysis showed that the rhizobiomes of *Z. marina* and *Z. noltii* within regions were highly similar, but strongly different from the rhizobiomes of the same species between regions (**Figure 4**).

The rhizosphere community composition of *Z. marina*, *Z. noltii*, and *C. nodosa* from Portugal was highly similar both at the phylum and class level (PERMANOVA, *P* = 0.1067 and *P* = 0.0706, respectively). *Proteobacteria* was the most abundant phylum among all three species, representing 65–68% of all OTUs. Other dominant phyla were *Bacteroidetes*, which accounted for 10–12%, *Chloroflexi* (4–5%), *Planctomycetes* (2–3.5%), *Actinobacteria* (2.5–3%), and *Acidobacteria* (2.2–2.6%). The most abundant classes detected were *Gammaproteobacteria* (32–38%) and *Deltaproteobacteria* (23–26%) from the phylum *Proteobacteria*, followed by *Bacteroidia* (*Bacteroidetes*, 6–7%), *Epsilonproteobacteria* (*Proteobacteria*, 2.7–4.4%), *Anaerolineae* (*Chloroflexi*, 4%), *Acidimicrobiia* (*Actinobacteria*, 2–2.6%), and *Alphaproteobacteria* (*Proteobacteria*, 1.5–2.7%).

The rhizobiomes of *Z. marina* and *Z. noltii* from France did not differ at the phylum level (PERMANOVA, *P* = 0.0166). *Proteobacteria* were dominant in the rhizobiomes of both seagrass species (46.6 and 41%, respectively) followed by *Bacteroidetes* (27.4 and 22.8%), *Firmicutes* (6.7 and 9%) and *Actinobacteria* (5 and 6.9%). At the class level, however, there were some differences in relative abundances (PERMANOVA, *P* = 0.0077). SIMPER analysis revealed that the *Epsilonproteobacteria* was the main taxon responsible for the differences observed between these two seagrasses in France, contributing 21.9% to the observed variation, followed by *Flavobacteriia* (8.6%), *Gammaproteobacteria* (7.5%), *Deltaproteobacteria* (7.3%), *Alphaproteobacteria* (7.3%), and *Clostridia* (7.2%). For a complete overview of the relative abundance of individual taxa at the phylum and class levels, see Supplementary Tables S1 and S2, respectively. Furthermore, the classes contributing the most for the differences observed between Portugal and France were dominated by families typically involved in the sulfur cycle (Supplementary Table S2).

### Core Seagrass Rhizobiome

**FIGURE 5:**
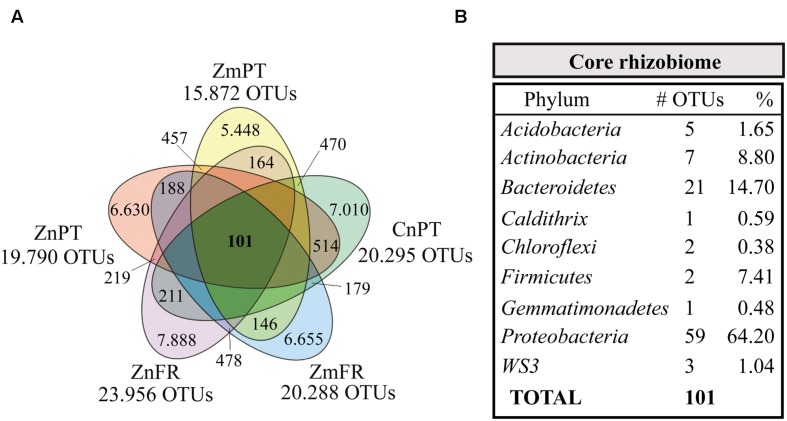
**Venn diagram showing the core rhizobiome of all seagrasses studied. (A)** *Zostera marina* (Zm-), *Z. noltii* (Zn-), both from Portugal (-PT) and France (-FR), and *C. nodosa* from Portugal (CnPT). The total number of OTUs clustered at a similarity level of 97% is represented under the label of each sample. The number of unique OTUs and OTUs shared between each combination of two samples is also shown. **(B)** Summarized taxonomic composition of the core rhizobiome, and respective number of OTUs present in each phylum. The relative number of the OTUs present in each phylum is also shown, indicated as percentage of sequences. A complete overview of the identity of the 101 OTUs that comprise the core rhizobiome of seagrasses is presented in Supplementary Table S3.

The bacterial communities of the different seagrass species were compared in order to reveal a core rhizobiome comprehending the OTUs present in all the rhizospheres (**Figure 5**, Supplementary Table S3). Only 0.2% of the OTUs and 15% of sequences present in all the samples were identified as core rhizobiome, and included members from the phyla *Proteobacteria*, *Bacteroidetes*, *Actinobacteria*, *Acidobacteria*, *Firmicutes*, WS3, *Chloroflexi*, *Caldithrix*, and *Gemmatimonadetes* (Supplementary Table S3). Sixty-four percent of the core taxa belonged to the *Proteobacteria*, with representatives of the classes *Alpha-*, *Gamma-, Delta-*, and *Epsilonproteobacteria* (Supplementary Table S3).

## Discussion

In this study, we evaluated differences between the microbial community structure of the rhizosphere of three seagrass species and their surrounding environment, as well as between two distant geographic locations. Rhizosphere, rhizoplane and endophytic compartments are different niches that host distinct microbial communities around, on and inside root surfaces, respectively. Here, we document the lack of significant differences between the rhizosphere microbiome of different seagrass species from one location. However it is noteworthy that further investigation into the composition of rhizoplane and endophytic compartments can possibly result in differences between seagrass species (Bulgarelli et al., 2012; Lundberg et al., 2012). The intimacy of the association between plant and microbe is expected to rise with increased proximity to the roots, due to direct contact with the plant and stronger exposure to its exudates (Bulgarelli et al., 2012; Lundberg et al., 2012).

### Rhizobiomes of Different Seagrasses Within and across Regions

Microbial assemblages in the rhizosphere of terrestrial plants have been shown to be shaped, for instance, by plant species (Berg and Smalla, 2009) and host genotype (Lundberg et al., 2012), and to vary according to the developmental stage of the plant (Chaparro et al., 2014) and type of soil/sediment (e.g., Berg and Smalla, 2009; Lundberg et al., 2012; Tkacz et al., 2015). It has also been demonstrated that microbial activity is sensitive to seasonal changes in seagrass beds (Smith et al., 2004). The type of soil and its particular physico-chemical characteristics have been repeatedly named as determinant for the community structure in the rhizosphere of terrestrial plants, while at the same time plants also influence soil properties (Philippot et al., 2013). Recently, Tkacz et al. (2015) concluded that the rhizobiome is controlled by soil composition along with plant type. Even though there is a vast amount of information that attributes differences in rhizobiomes to the type of soil/sediment, to our knowledge there is no information regarding the identity of their particular properties. Because grain size, organic matter and water content directly affect sediment’s microbial communities and plant morphology (Wicks et al., 2009), they were used in this study as indicators of different types of sediment. Our results highlight that the grain size of *Z. marina* sediments in Portugal was significantly smaller than that of sediments inhabited by the other two seagrasses (very fine sand vs. medium sand, respectively), which corresponded with the higher organic matter and water contents observed in the sediments of this species. Fine sediments tend to have a higher water retention capacity, which in turn allows organic matter to be more strongly trapped (Langston and Ridgway, 2006). Given our results, we consider that for the study area at Culatra island, sediment type, plant species, or even a shared effect of both, are not determinant for the identity of the rhizobiomes.

The rhizobiome class composition of seagrasses in France described in this study corresponds well with that previously reported for a *Zostera noltii*-colonized sediment from Bassin d’Arcachon, Southwestern France, which was dominated by *Deltaproteobacteria* (36%) and *Gammaproteobacteria* (27%) based on 16S rRNA gene cloning (Cifuentes et al., 2000). When comparing the rhizobiomes of *Zostera* spp. between Portugal and France, we observed significant differences between both locations. According to the hologenome theory of evolution, the diverse and abundant consortium of microorganisms evolves in association with their host as one single entity, but is susceptible to (fast) variations if a change is brought into the system via host genome or microbiome (Rosenberg et al., 2007; Zilber-Rosenberg and Rosenberg, 2008). On sight of our results, we consider that the rhizobiome is a part of the seagrass holobiont, and that at a local scale its selection is driven by the host, whereas at large scale/long distance, the selection is more strongly shaped by the environment. This is supported by the presence of similar rhizobiomes within Portugal, although their sediment characteristics significantly differ, concomitantly with the presence of differences between the rhizobiomes of Portugal and France, despite the similarities in their sediment characteristics.

### Core Seagrass Rhizobiome

The top five most abundant core OTUs (abundance between 5 and 10% of the core rhizobiome) were classified as members of five different classes, *Epsilonproteobacteria* (family *Helicobacteraceae*), *Acidimicrobiia* (family koll13), *Gammaproteobacteria* (order *Chromatiales*), *Deltaproteobacteria* (genus *Desulfococcus*), and *Clostridia* (order *Clostridiales*). Likewise, *Gamma-* and *Deltaproteobacteria* have previously been found to be among the most abundant members of the core microbiome of belowground structures in the seagrass *Halophila stipulacea* (Mejia et al., 2016). The *Clostridiales* and other core taxa belonging to the alphaproteobacterial family *Rhodobacteraceae* (Meyer and Kuever, 2007), the *Delta-* (Muyzer and Stams, 2008) and *Epsilonproteobacteria* (Campbell et al., 2006) classes, as well as the gammaproteobacterial orders *Thiotrichales* (Garrity et al., 2005) and *Alteromonadales* (Fuchs et al., 2007) are likely to be involved in sulfur processes, such as sulfate reduction and sulfur oxidation. Members of the order *Clostridiales* (*Firmicutes*), for instance, can be involved in sulfate reduction (*Desulfotomaculum* sp.; Widdel, 2006), as well as in processes such as nitrogen fixation (e.g., *Clostridium pasteurianum*; Chen, 2004) and fermentation (e.g., *Clostridium acetobutylicum*; Cato et al., 1986). One of the fermentation products of solventogenic *Clostridia* is acetone (Han et al., 2011), which can be completely oxidized by other bacteria, such as the sulfate-reducing *Desulfococcus biacutus* (Platen et al., 1990). In the present work, the genus *Desulfococcus* was one of the most abundant genera (specifically 7%, Supplementary Table S3). This genus was represented by 5 different OTUs and although the taxonomic resolution of some of the core OTUs was very low, the data indicated this as the most diverse genus in the core. Members of *Desulfococcus* are important hydrocarbon degraders (Miralles et al., 2007; Apostolopoulou et al., 2014). Their high abundance and diversity in the core rhizobiome of seagrasses might function as a buffer between contaminated sediments and the plants, thereby detoxifying the root area from these phytotoxic compounds (Saxe, 1996).

Comprising 21 OTUs and ranking as the second most diverse and abundant phylum (14.7%) in the core seagrass rhizobiome are the *Bacteroidetes* that are known to be widespread across marine environments, including sediments (e.g., Khan et al., 2007). Within this phylum, the cluster *Cytophaga-Flavobacteria* is highly abundant and one of the most represented groups in aquatic ecosystems (Kirchman, 2002). They are important decomposers of high molecular weight organic matter (Kirchman, 2002), such as cellulose and chitin (Fenchel, 2012).

*Actinobacteria* are common inhabitants of roots of terrestrial plants, where they might function as biocontrol agents against pathogens (e.g., Ting et al., 2009). However, little is known about their presence and function in the rhizosphere of seagrasses (Ravikumar et al., 2012; Wu et al., 2012; Jose et al., 2014). Here we found that 7 OTUs (9% of the core taxa present in the studied seagrasses) belonged to this phylum, possibly specifically recruited by the plant in order to provide protection against pathogens, therefore facilitating its immune responses (Cook et al., 1995; Mendes et al., 2011).

Although many functions of the abovementioned taxa have already been described, the ecological role of members of some of these phyla, such as *Acidobacteria*, *Caldithrix, Chloroflexi*, and *Gemmatimonadetes* to the seagrass rhizobiome is unclear. This is mainly due to the small number of strains that have been isolated and characterized. In some cases, their role is completely unknown, because cultured representatives are lacking, which is the case for members of the phylum WS3. Nevertheless, it is possible to infer putative roles of some members of these taxa. Members of WS3 for instance, have been consistently detected in sediments (e.g., Ikenaga et al., 2010; Zeng et al., 2010), and the genus KSB4, in particular, was previously identified in sulfide-rich sediments of coastal environments (Tanner et al., 2000), which might indicate a possible role in the sulfur cycle. The first cultured representative of the phylum *Gemmatimonadetes, Gemmatimonas aurantiaca*, was isolated from an anaerobic-aerobic sequential batch reactor used for enhanced biological phosphorus removal from wastewater. The strain could accumulate polyphosphate and so might be involved in the phosphorus cycle. DeBruyn et al. (2011) studied the ecology of the *Gemmatimonadetes* and found that it was ubiquitously present in soil. *Acidobacteria* are frequently abundant in sediments and soils, and although they have very few isolated members, the genome analysis of three strains from this phylum performed by Ward et al. (2009) suggests that these bacteria might be involved in the production of antimicrobial compounds, stabilization of the soil structure, and also in the degradation of complex sugars such as chitin, which highlights the possible importance of these bacteria in the carbon cycling in marine biomes.

The identification of the core rhizobiome provides insights into key host-microbe interactions, by detecting which bacteria consistently inhabit the rhizosphere, thereby stressing their potential importance in the maintenance of a healthy seagrass holobiont. Even though the percentage of some of the individual core OTUs was low (Supplementary Table S3), their activity is likely to be higher than more abundant OTUs, as previously reported in comparisons between rare biosphere and common OTUs from different environments (reviewed by Lynch and Neufeld, 2015). Identifying these taxa provides important cues for the understanding of the phylogenetic (and potentially functional) identity of the consortium of bacteria inhabiting the seagrass rhizosphere. By identifying the core microbiome among three seagrass species in two different locations, we covered the spatial dynamism regardless of differences caused by different “hosts” in a biogeographical framework. Moreover, the detection of genera like *Desulfococcus*, which harbored 5 different OTUs (clustered at 97% similarity) whereas only 2 species have been described in the literature (Kuever et al., 2005b), together with all the unclassified OTUs widespread among the remaining taxa, indicates that the rhizosphere recruits a widely unknown variety of microorganisms potentially essential for the plants.

The role of the core taxa identified in this study is likely to go beyond its importance for the seagrasses. Bacteria like *Clostridium* sp. (Wagner and Stadtman, 1962) and *Desulfovibrio* sp. (van der Maarel et al., 1996), both present in the core, are able to degrade the osmoprotectant dimethylsulfoniopropionate (DMSP) into dimethylsulfide (DMS; reviewed in Yoch, 2002). DMSP is produced by micro- and macroalgae, as well as by seagrasses, and is highly abundant in marine sediments (Jonkers et al., 2000). One of the end products of this cleavage process is DMS, which is a volatile organic sulfur compound that has the ability to control global climate by enhancing the albedo and formation of clouds in the atmosphere (Shaw, 1983; Charlson et al., 1987). Therefore, the core seagrass rhizobiome might play a pivotal role in climate change by harboring bacteria directly involved in DMS formation.

### Niche Differentiation of Sulfur Bacteria

**FIGURE 6:**
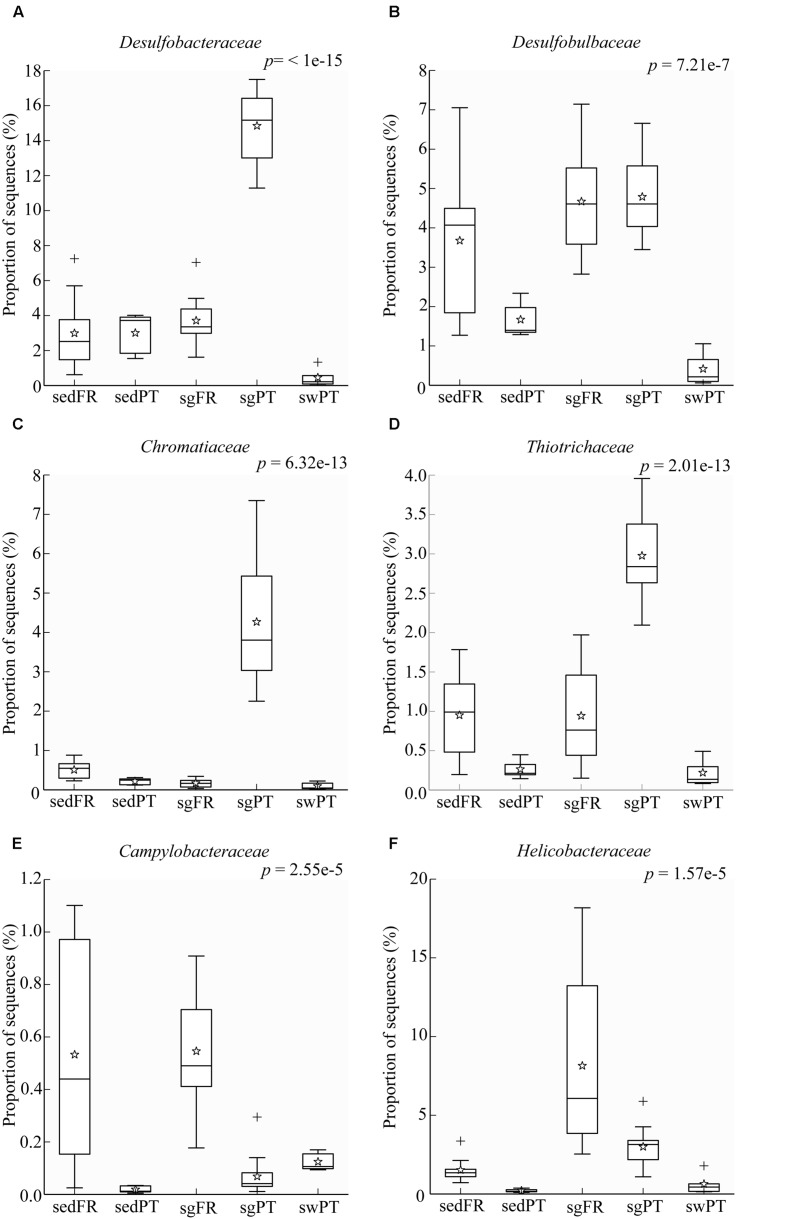
**Box plots showing the relative abundance of sequences of the most abundant bacterial groups involved in sulfur processes. (A)** *Desulfobacteraceae*, **(B)** *Desulfobulbaceae*, **(C)** *Chromatiaceae*, **(D)** *Thiotrichaceae*, **(E)** *Campylobacteraceae*, and **(F)** *Helicobacteraceae*. Indicated are bulk sediments from Portugal (sedPT) and France (sedFR); seagrasses from Portugal (sgPT) and France (sgFR); seawater from Portugal (swPT). The top of the box indicates the third quartile, the bottom the first quartile, and the line in the middle is the median. The star indicates the mean of the data, crosses are outliers, and the whiskers represent error bars.

Sulfate reduction is one of the main processes in marine sediments (Jørgensen, 1982), but in particular in seagrass meadows where it is fuelled by exudates from the plant roots (Isaksen and Finster, 1996; Holmer and Nielsen, 1997; Hansen et al., 2000). The class *Deltaproteobacteria* was represented predominantly by members of the family *Desulfobacteraceae* in the rhizobiomes of seagrasses collected from Portugal, and by *Desulfobulbaceae* in both France and Portugal (**Figures 6A,B**). The most dominant members of the *Desulfobacteraceae* in the rhizobiomes of seagrasses from Portugal were affiliated to the genus *Desulfococcus* and to a lesser extent to *Desulfosarcina.* Members of these genera use sulfate, sulfite, and thiosulfate as electron acceptors to oxidize different fatty acids and alcohols completely to CO_2_ (Brysch et al., 1987). The dominant members within the *Desulfobulbaceae* were uncultured lineages and *Desulfocapsa.* In general, members belonging to the *Desulfobulbaceae* use sulfate, sulfite, and thiosulfate as electron acceptors to oxidize different fatty acids incompletely to acetate (Kuever et al., 2005c). In addition, *Desulfocapsa* can also disproportionate thiosulfate and elemental sulfur to H_2_S and sulfate (Finster et al., 1998). Like genera belonging to the *Desulfobacteraceae* family, *Desulfocapsa* are also able to oxidize alcohols. During the night cycle, the lack of oxygen around the roots leads seagrass root tissues to switch to fermentation, which causes the release of ethanol to the rhizosphere (Smith et al., 1988). This association might represent a fair trade between host and microbe, in which during the night bacteria use ethanol as electron donor to remove this alcohol from the surrounding of the roots (Kuever et al., 2005a) although during light conditions they release hydrogen sulfide. The versatility of bacteria able to perform both sulfate reduction and elemental sulfur and thiosulfate disproportionation can be a very advantageous feature to the seagrass-microbe interactions occurring in the rhizosphere.

Sulfide, which is highly toxic to seagrasses, can be neutralized by the activity of sulfur-oxidizing bacteria. Joshi and Hollis (1977) described the protection of rice seedlings from H_2_S by the colorless sulfur-oxidizing gammaproteobacterium *Beggiatoa.* Recently, van der Heide et al. (2012) nicely demonstrated that sulfide-oxidizing bacteria in the gills of the lucinid bivalve *Loripes lacteus* could reduce sulfide stress for the seagrass *Zostera noltii.* However, they also found that *Z. noltii* without bivalves could to some extent also reduce the sulfide concentration, which might be the result of the seagrass (Hasler-Sheetal and Holmer, 2015) and indigenous sulfur-oxidizing bacteria in the rhizosphere of the seagrass. In our sediments, *Loripes* or related lucinid bivalves were absent, and so sulfur-oxidizing bacteria of the *Gamma*- and/or *Epsilonproteobacteria* probably contributed to detoxification of sulfide. These bacteria were represented by *Chromatiaceae* and *Thiotrichaceae* of the *Gammaproteobacteria* in the rhizobiomes of seagrasses from Portugal (**Figures 6C,D**), while members of the *Campylobacteraceae* and *Helicobacteraceae* both belonging to the *Epsilonproteobacteria* were mainly found in the rhizobiomes of seagrasses collected in France (**Figures 6E,F**). Although *Chromatiaceae* are predominantly phototrophic, some of them might be able to colonize the rhizosphere by switching their growth strategy to chemo-, organo-, and/or mixotrophy, according to the environmental conditions (e.g., Madigan and Gest, 1979; Kämpf and Pfennig, 1980). The most dominant genus within the *Helicobacteraceae* was *Sulfurimonas*, while members of the genera *Arcobacter* and *Sulfurospirillum*, both belonging to the *Campylobacteraceae*, were present at lower numbers. Similar results were obtained by Jensen et al. (2007), who found a dominance of sulfur-oxidizing bacteria affiliated to the *Epsilonproteobacteria*, such as *Arcobacter and Sulfurimonas* in the rhizosphere of *Z. marina* in Denmark. Thomas et al. (2014), who studied the abundance and activity of sulfur-oxidizing bacteria in salt marsh sediments colonized by the plant *Spartina alterniflora*, found a predominance of sulfur-oxidizing bacteria of the *Gammaproteobacteria* (*Chromatiales* and *Thiotrichales*) and to a lesser extend *Alpha-* and *Epsilonproteobacteria.*

This study shows that the seagrass rhizosphere is dominated by bacteria involved in the sulfur cycle, and it strongly suggests that, regardless their phylogenetic affiliation, the functionality of bacteria related to sulfur processes is maintained across different regions. At a small scale/short distance, seagrass rhizobiomes are shaped by the plant (although not at a host species level), and at a large scale/long distance level, they are shaped by environmental differences, extrinsic to plant phylogeny. Furthermore, our results suggest that sediment grain size and percentage of organic matter are not determinant for the microbial structure of the seagrass rhizobiomes at Culatra island, therefore further (experimental) research is required in order to reveal the parameters exerting the environmental pressure observed across regions.

## Author Contributions

CC, AE, and GM designed the study and collected the samples. CC performed lab-experiments and data analysis. CC wrote the manuscript and all authors contributed to the discussion of the results and to the final version of the manuscript.

## Conflict of Interest Statement

The authors declare that the research was conducted in the absence of any commercial or financial relationships that could be construed as a potential conflict of interest.
