# Supplementary material for: Rhizosphere Microbiomes of European Seagrasses Are Selected by the Plant, But Are Not Species Specific
Source: Front Microbiol. 2016 Mar 31;7:440. doi: 10.3389/fmicb.2016.00440 (PMC4815253; doi:10.3389/fmicb.2016.00440)
Supplement: Supplementary file 1 [file Table_1.DOCX]

Supplementary Table S1

Rhizosphere microbiomes of European seagrasses are selected by the plant, but are not species specific

Catarina Cúcio, Aschwin H. Engelen, Rodrigo Costa, Gerard Muyzer*

***Correspondence:** g.muijzer@uva.nl

Supplementary Table S1 – Average percentage of phyla present in all rhizospheres of *Z, marina*, *Z, noltii* and *C, nodosa* as well as in the bulk sediment and seawater collected in Portugal (Zm-, Zn, Cn-, Sed- and SwPT, respectively), and rhizospheres from *Z, marina* and *Z, noltii* (Zm-and ZnFR, respectively) and respective bulk sediments (SedM and SedN) collected in France.

| Taxon | ZmPT | ZnPT | CnPT | SedPT | SwPT | ZmFR | ZnFR | SedM | SedM |
| --- | --- | --- | --- | --- | --- | --- | --- | --- | --- |
| Other | 0,587 | 0,667 | 0,812 | 0,783 | 0,241 | 1,049 | 1,518 | 0,417 | 0,808 |
| Unknown Bacteria | 0,014 | 0,013 | 0,030 | 0,009 | 0,000 | 0,011 | 0,020 | 0,004 | 0,011 |
| AC1 | 0,022 | 0,016 | 0,036 | 0,001 | 0,001 | 0,001 | 0,041 | 0,000 | 0,033 |
| Acidobacteria | 2,248 | 2,407 | 2,588 | 3,152 | 0,068 | 1,051 | 1,575 | 1,150 | 1,793 |
| Actinobacteria | 2,454 | 2,709 | 3,049 | 5,135 | 1,858 | 5,095 | 6,861 | 11,021 | 5,562 |
| AncK6 | 0,000 | 0,000 | 0,000 | 0,003 | 0,000 | 0,000 | 0,000 | 0,000 | 0,000 |
| Armatimonadetes | 0,000 | 0,000 | 0,000 | 0,001 | 0,000 | 0,000 | 0,000 | 0,001 | 0,001 |
| BHI80-139 | 0,001 | 0,002 | 0,002 | 0,000 | 0,000 | 0,000 | 0,000 | 0,000 | 0,000 |
| BRC1 | 0,036 | 0,077 | 0,088 | 0,108 | 0,002 | 0,050 | 0,032 | 0,030 | 0,058 |
| Bacteroidetes | 11,715 | 11,593 | 9,571 | 8,012 | 26,945 | 27,384 | 22,811 | 24,263 | 17,852 |
| Caldiserica | 0,001 | 0,002 | 0,000 | 0,000 | 0,000 | 0,019 | 0,018 | 0,003 | 0,004 |
| Caldithrix | 0,503 | 0,420 | 0,914 | 0,563 | 0,012 | 0,098 | 0,416 | 0,036 | 0,372 |
| Chlamydiae | 0,010 | 0,005 | 0,032 | 0,107 | 0,002 | 0,018 | 0,092 | 0,027 | 0,048 |
| Chlorobi | 0,096 | 0,130 | 0,184 | 0,231 | 0,018 | 0,295 | 0,667 | 0,139 | 0,571 |
| Chloroflexi | 4,695 | 4,389 | 4,946 | 1,500 | 0,114 | 0,688 | 1,853 | 0,282 | 1,189 |
| Cyanobacteria | 0,063 | 0,132 | 0,065 | 0,465 | 0,061 | 0,521 | 0,374 | 1,941 | 1,997 |
| Deferribacteres | 0,000 | 0,002 | 0,000 | 0,000 | 0,000 | 0,001 | 0,000 | 0,000 | 0,000 |
| Elusimicrobia | 0,064 | 0,075 | 0,057 | 0,034 | 0,003 | 0,011 | 0,011 | 0,002 | 0,013 |
| FCPU426 | 0,003 | 0,001 | 0,008 | 0,000 | 0,000 | 0,002 | 0,001 | 0,001 | 0,004 |
| Fibrobacteres | 0,259 | 0,369 | 0,495 | 0,090 | 0,057 | 0,047 | 0,251 | 0,042 | 0,075 |
| Firmicutes | 0,579 | 0,535 | 0,704 | 0,557 | 0,216 | 6,712 | 9,094 | 2,724 | 2,649 |
| Fusobacteria | 0,046 | 0,043 | 0,043 | 0,065 | 0,035 | 0,044 | 0,087 | 0,503 | 2,732 |
| GN02 | 0,062 | 0,083 | 0,070 | 0,090 | 0,081 | 0,237 | 0,405 | 0,126 | 0,271 |
| GN04 | 0,095 | 0,099 | 0,221 | 0,079 | 0,001 | 0,027 | 0,174 | 0,007 | 0,144 |
| GOUTA4 | 0,000 | 0,000 | 0,002 | 0,002 | 0,000 | 0,000 | 0,013 | 0,000 | 0,005 |
| Gemmatimonadetes | 0,878 | 1,061 | 1,059 | 1,521 | 0,054 | 0,432 | 0,716 | 0,317 | 0,820 |
| H-178 | 0,016 | 0,023 | 0,022 | 0,001 | 0,009 | 0,022 | 0,051 | 0,007 | 0,034 |
| Hyd24-12 | 0,016 | 0,009 | 0,019 | 0,000 | 0,002 | 0,028 | 0,078 | 0,005 | 0,021 |
| KSB3 | 0,114 | 0,120 | 0,235 | 0,017 | 0,010 | 0,034 | 0,158 | 0,032 | 0,086 |
| Kazan-3B-28 | 0,000 | 0,000 | 0,000 | 0,003 | 0,000 | 0,000 | 0,002 | 0,000 | 0,001 |
| LCP-89 | 0,053 | 0,069 | 0,075 | 0,002 | 0,002 | 0,027 | 0,052 | 0,002 | 0,047 |
| LD1 | 0,006 | 0,002 | 0,007 | 0,001 | 0,000 | 0,000 | 0,008 | 0,000 | 0,010 |
| Lentisphaerae | 0,191 | 0,388 | 0,393 | 0,402 | 0,075 | 0,096 | 0,087 | 0,086 | 0,225 |
| MVS-104 | 0,002 | 0,007 | 0,013 | 0,000 | 0,000 | 0,000 | 0,001 | 0,000 | 0,004 |
| NKB19 | 0,014 | 0,023 | 0,030 | 0,099 | 0,001 | 0,036 | 0,039 | 0,022 | 0,042 |
| Nitrospirae | 0,049 | 0,076 | 0,084 | 0,810 | 0,001 | 0,004 | 0,012 | 0,010 | 0,145 |
| OD1 | 0,110 | 0,147 | 0,172 | 0,302 | 0,188 | 0,515 | 0,773 | 0,327 | 0,462 |
| OP1 | 0,007 | 0,002 | 0,005 | 0,014 | 0,000 | 0,001 | 0,010 | 0,001 | 0,007 |
| OP11 | 0,000 | 0,000 | 0,000 | 0,001 | 0,000 | 0,000 | 0,000 | 0,000 | 0,000 |
| OP3 | 0,113 | 0,121 | 0,161 | 0,159 | 0,031 | 0,014 | 0,055 | 0,016 | 0,063 |
| OP8 | 0,328 | 0,269 | 0,452 | 0,008 | 0,011 | 0,068 | 0,216 | 0,023 | 0,231 |
| OP9 | 0,000 | 0,000 | 0,000 | 0,000 | 0,000 | 0,000 | 0,000 | 0,000 | 0,000 |
| PAUC34f | 0,002 | 0,002 | 0,003 | 0,003 | 0,000 | 0,000 | 0,002 | 0,001 | 0,004 |
| Planctomycetes | 2,287 | 3,022 | 3,447 | 4,582 | 0,235 | 1,794 | 2,247 | 2,316 | 2,572 |
| Poribacteria | 0,000 | 0,000 | 0,000 | 0,002 | 0,000 | 0,000 | 0,000 | 0,000 | 0,000 |
| Proteobacteria | 68,321 | 65,849 | 64,554 | 66,283 | 69,128 | 46,640 | 41,069 | 49,487 | 53,646 |
| SAR406 | 0,041 | 0,072 | 0,060 | 0,057 | 0,011 | 0,064 | 0,214 | 0,019 | 0,070 |
| SBR1093 | 0,000 | 0,002 | 0,000 | 0,175 | 0,000 | 0,002 | 0,007 | 0,006 | 0,023 |
| SR1 | 0,002 | 0,004 | 0,002 | 0,002 | 0,004 | 0,062 | 0,056 | 0,046 | 0,021 |
| Spirochaetes | 1,024 | 1,200 | 1,675 | 0,404 | 0,150 | 0,792 | 0,969 | 0,312 | 0,711 |
| TM6 | 0,018 | 0,024 | 0,044 | 0,080 | 0,010 | 0,033 | 0,165 | 0,021 | 0,076 |
| TM7 | 0,013 | 0,011 | 0,019 | 0,039 | 0,029 | 0,116 | 0,122 | 0,234 | 0,118 |
| TPD-58 | 0,000 | 0,001 | 0,007 | 0,001 | 0,000 | 0,001 | 0,010 | 0,000 | 0,002 |
| Tenericutes | 0,013 | 0,042 | 0,046 | 0,042 | 0,043 | 0,040 | 0,038 | 0,023 | 0,033 |
| Verrucomicrobia | 1,328 | 1,947 | 1,603 | 3,033 | 0,224 | 4,411 | 3,711 | 3,342 | 2,592 |
| WPS-2 | 0,000 | 0,002 | 0,000 | 0,000 | 0,002 | 0,005 | 0,011 | 0,029 | 0,052 |
| WS2 | 0,023 | 0,018 | 0,027 | 0,051 | 0,001 | 0,051 | 0,157 | 0,026 | 0,083 |
| WS3 | 1,420 | 1,653 | 1,779 | 0,841 | 0,050 | 1,108 | 2,175 | 0,514 | 1,444 |
| WS5 | 0,001 | 0,000 | 0,002 | 0,000 | 0,001 | 0,000 | 0,000 | 0,000 | 0,000 |
| WWE1 | 0,011 | 0,022 | 0,027 | 0,000 | 0,007 | 0,216 | 0,447 | 0,012 | 0,087 |
| ZB3 | 0,005 | 0,006 | 0,008 | 0,007 | 0,003 | 0,001 | 0,006 | 0,000 | 0,013 |
| [Caldithrix] | 0,042 | 0,038 | 0,052 | 0,038 | 0,004 | 0,006 | 0,015 | 0,002 | 0,019 |
| [Thermi] | 0,000 | 0,001 | 0,000 | 0,035 | 0,000 | 0,020 | 0,010 | 0,045 | 0,044 |
