# Supplementary material for: Rhizosphere Microbiomes of European Seagrasses Are Selected by the Plant, But Are Not Species Specific
Source: Front Microbiol. 2016 Mar 31;7:440. doi: 10.3389/fmicb.2016.00440 (PMC4815253; doi:10.3389/fmicb.2016.00440)
Supplement: Supplementary file 2 [file Table_2.DOCX]

Supplementary Table S2

Rhizosphere microbiomes of European seagrasses are selected by the plant, but are not species specific

Catarina Cúcio, Aschwin H. Engelen, Rodrigo Costa, Gerard Muyzer*

*** Correspondence:** g.muijzer@uva.nl

Supplementary Table S2 – Average percentage of classes present in all rhizospheres of *Z, marina*, *Z, noltii* and *C, nodosa* as well as in the bulk sediment and seawater collected in Portugal (Zm-, Zn, Cn-, Sed- and SwPT, respectively), and rhizospheres from *Z, marina* and *Z, noltii* (Zm-and ZnFR, respectively) and respective bulk sediments (SedM and SedN) collected in France.

| Taxon | ZmPT | | ZnPT | CnPT | SedPT | SwPT | | ZmFR | | ZnFR | SedM | SedN | |
| --- | --- | --- | --- | --- | --- | --- | --- | --- | --- | --- | --- | --- | --- |
| Other; Other | 0,587 | | 0,667 | 0,008 | 0,783 | 0,241 | | 1,049 | | 0,015 | 0,417 | 0,808 | |
| Unknown Bacteria | 0,014 | | 0,013 | 0,000 | 0,009 | 0,000 | | 0,011 | | 0,000 | 0,004 | 0,011 | |
| AC1; Other | 0,002 | | 0,003 | 0,000 | 0,000 | 0,001 | | 0,000 | | 0,000 | 0,000 | 0,000 | |
| AC1; HDBW-WB69 | 0,006 | | 0,008 | 0,000 | 0,001 | 0,000 | | 0,000 | | 0,000 | 0,000 | 0,006 | |
| AC1; SHA-114 | 0,014 | | 0,005 | 0,000 | 0,000 | 0,001 | | 0,001 | | 0,000 | 0,000 | 0,027 | |
| Acidobacteria; Other | 0,002 | | 0,009 | 0,000 | 0,019 | 0,000 | | 0,003 | | 0,000 | 0,001 | 0,015 | |
| Acidobacteria | 0,041 | | 0,044 | 0,001 | 0,208 | 0,001 | | 0,012 | | 0,000 | 0,003 | 0,038 | |
| Acidobacteria; AT-s2-57 | 0,149 | | 0,163 | 0,001 | 0,080 | 0,001 | | 0,018 | | 0,000 | 0,018 | 0,052 | |
| Acidobacteria; AT-s54 | 0,003 | | 0,003 | 0,000 | 0,068 | 0,001 | | 0,007 | | 0,000 | 0,023 | 0,015 | |
| Acidobacteria; Acidobacteria-6 | 0,140 | | 0,167 | 0,001 | 0,102 | 0,003 | | 0,064 | | 0,001 | 0,065 | 0,118 | |
| Acidobacteria; BPC102 | 0,017 | | 0,017 | 0,000 | 0,004 | 0,000 | | 0,000 | | 0,000 | 0,000 | 0,010 | |
| Acidobacteria; Holophagae | 0,013 | | 0,022 | 0,000 | 0,032 | 0,000 | | 0,017 | | 0,000 | 0,008 | 0,021 | |
| Acidobacteria; OS-K | 0,969 | | 0,970 | 0,009 | 0,160 | 0,026 | | 0,596 | | 0,010 | 0,420 | 0,695 | |
| Acidobacteria; RB25 | 0,356 | | 0,332 | 0,006 | 0,527 | 0,010 | | 0,144 | | 0,003 | 0,111 | 0,538 | |
| Acidobacteria; Solibacteres | 0,046 | | 0,053 | 0,001 | 0,413 | 0,000 | | 0,049 | | 0,000 | 0,015 | 0,037 | |
| Acidobacteria; Sva0725 | 0,506 | | 0,623 | 0,006 | 1,354 | 0,025 | | 0,099 | | 0,001 | 0,314 | 0,205 | |
| Acidobacteria; [Chloracidobacteria] | 0,002 | | 0,004 | 0,000 | 0,125 | 0,001 | | 0,042 | | 0,000 | 0,170 | 0,047 | |
| Acidobacteria; iii1-8 | 0,002 | | 0,001 | 0,000 | 0,059 | 0,000 | | 0,001 | | 0,000 | 0,000 | 0,002 | |
| Actinobacteria; Other | 0,001 | | 0,007 | 0,000 | 0,028 | 0,002 | | 0,019 | | 0,000 | 0,041 | 0,030 | |
| Actinobacteria | 0,001 | | 0,001 | 0,000 | 0,000 | 0,001 | | 0,000 | | 0,000 | 0,000 | 0,000 | |
| Actinobacteria; Acidimicrobiia | 2,091 | | 2,193 | 0,026 | 4,984 | 0,648 | | 4,945 | | 0,066 | 10,888 | 5,396 | |
| Actinobacteria; Actinobacteria | 0,222 | | 0,323 | 0,002 | 0,069 | 1,193 | | 0,064 | | 0,002 | 0,036 | 0,079 | |
| Actinobacteria; MB-A2-108 | 0,000 | | 0,000 | 0,000 | 0,002 | 0,000 | | 0,000 | | 0,000 | 0,000 | 0,000 | |
| Actinobacteria; Nitriliruptoria | 0,000 | | 0,003 | 0,000 | 0,012 | 0,000 | | 0,000 | | 0,000 | 0,003 | 0,000 | |
| Actinobacteria; OPB41 | 0,099 | | 0,113 | 0,001 | 0,008 | 0,009 | | 0,045 | | 0,000 | 0,025 | 0,035 | |
| Actinobacteria; Thermoleophilia | 0,040 | | 0,069 | 0,001 | 0,031 | 0,006 | | 0,021 | | 0,000 | 0,028 | 0,022 | |
| AncK6 | 0,000 | | 0,000 | 0,000 | 0,003 | 0,000 | | 0,000 | | 0,000 | 0,000 | 0,000 | |
| Armatimonadetes; [Fimbriimonadia] | 0,000 | | 0,000 | 0,000 | 0,001 | 0,000 | | 0,000 | | 0,000 | 0,001 | 0,001 | |
| BHI80-139; MD2896-B26 | 0,001 | | 0,002 | 0,000 | 0,000 | 0,000 | | 0,000 | | 0,000 | 0,000 | 0,000 | |
| BRC1; Other | 0,000 | | 0,001 | 0,000 | 0,000 | 0,000 | | 0,000 | | 0,000 | 0,000 | 0,000 | |
| BRC1 | 0,005 | | 0,008 | 0,000 | 0,011 | 0,000 | | 0,000 | | 0,000 | 0,000 | 0,010 | |
| BRC1; NPL-UPA2 | 0,005 | | 0,009 | 0,000 | 0,028 | 0,001 | | 0,023 | | 0,000 | 0,021 | 0,017 | |
| BRC1; PRR-11 | 0,027 | | 0,059 | 0,001 | 0,069 | 0,001 | | 0,027 | | 0,000 | 0,009 | 0,032 | |
| Bacteroidetes; Other | 0,899 | | 0,880 | 0,009 | 0,223 | 0,225 | | 1,729 | | 0,019 | 0,704 | 0,738 | |
| Bacteroidetes; At12OctB3 | 0,012 | | 0,022 | 0,000 | 0,002 | 0,001 | | 0,017 | | 0,000 | 0,002 | 0,004 | |
| Bacteroidetes; BME43 | 0,002 | | 0,003 | 0,000 | 0,027 | 0,003 | | 0,043 | | 0,000 | 0,085 | 0,096 | |
| Bacteroidetes; Bacteroidia | 7,307 | | 6,430 | 0,060 | 1,363 | 0,444 | | 7,669 | | 0,068 | 1,283 | 2,051 | |
| Bacteroidetes; Cytophagia | 1,242 | | 1,525 | 0,010 | 1,274 | 0,104 | | 0,638 | | 0,006 | 0,359 | 0,596 | |
| Bacteroidetes; Flavobacteriia | 1,881 | | 2,267 | 0,014 | 2,813 | 25,742 | | 14,303 | | 0,114 | 17,397 | 10,485 | |
| Bacteroidetes; Sphingobacteriia | 0,011 | | 0,013 | 0,000 | 0,091 | 0,266 | | 0,153 | | 0,000 | 0,223 | 0,086 | |
| Bacteroidetes; [Rhodothermi] | 0,033 | | 0,037 | 0,000 | 0,484 | 0,008 | | 0,102 | | 0,001 | 0,288 | 0,198 | |
| Bacteroidetes; [Saprospirae] | 0,328 | | 0,416 | 0,003 | 1,734 | 0,152 | | 2,730 | | 0,019 | 3,921 | 3,597 | |
| Caldiserica; OP5 | 0,001 | | 0,002 | 0,000 | 0,000 | 0,000 | | 0,019 | | 0,000 | 0,003 | 0,004 | |
| Caldithrix; Caldithrixae | 0,503 | | 0,420 | 0,009 | 0,563 | 0,012 | | 0,098 | | 0,004 | 0,036 | 0,372 | |
| Chlamydiae; Chlamydiia | 0,010 | | 0,005 | 0,000 | 0,107 | 0,002 | | 0,018 | | 0,001 | 0,027 | 0,048 | |
| Chlorobi | 0,012 | | 0,017 | 0,000 | 0,011 | 0,002 | | 0,015 | | 0,001 | 0,002 | 0,012 | |
| Chlorobi; BSV26 | 0,000 | | 0,000 | 0,000 | 0,019 | 0,000 | | 0,000 | | 0,000 | 0,000 | 0,002 | |
| Chlorobi; Ignavibacteria | 0,075 | | 0,082 | 0,001 | 0,150 | 0,009 | | 0,257 | | 0,005 | 0,108 | 0,482 | |
| Chlorobi; OPB56 | 0,008 | | 0,029 | 0,000 | 0,050 | 0,007 | | 0,024 | | 0,001 | 0,029 | 0,071 | |
| Chlorobi; SJA-28 | 0,001 | | 0,002 | 0,000 | 0,000 | 0,000 | | 0,000 | | 0,000 | 0,000 | 0,004 | |
| Chloroflexi; Other | 0,006 | | 0,008 | 0,000 | 0,005 | 0,002 | | 0,003 | | 0,000 | 0,001 | 0,004 | |
| Chloroflexi; Anaerolineae | 4,195 | | 3,784 | 0,041 | 0,622 | 0,077 | | 0,572 | | 0,016 | 0,230 | 0,943 | |
| Chloroflexi; Dehalococcoidetes | 0,178 | | 0,254 | 0,004 | 0,012 | 0,009 | | 0,009 | | 0,001 | 0,000 | 0,139 | |
| Chloroflexi; Ellin6529 | 0,300 | | 0,324 | 0,003 | 0,138 | 0,024 | | 0,100 | | 0,001 | 0,048 | 0,083 | |
| Chloroflexi; Ktedonobacteria | 0,003 | | 0,000 | 0,000 | 0,000 | 0,000 | | 0,000 | | 0,000 | 0,000 | 0,000 | |
| Chloroflexi; MVP-21 | 0,000 | | 0,000 | 0,000 | 0,000 | 0,000 | | 0,001 | | 0,000 | 0,001 | 0,000 | |
| Chloroflexi; S085 | 0,001 | | 0,000 | 0,000 | 0,122 | 0,000 | | 0,000 | | 0,000 | 0,000 | 0,000 | |
| Chloroflexi; SAR202 | 0,014 | | 0,018 | 0,000 | 0,470 | 0,001 | | 0,000 | | 0,000 | 0,001 | 0,004 | |
| Chloroflexi; TK17 | 0,000 | | 0,000 | 0,000 | 0,106 | 0,000 | | 0,002 | | 0,000 | 0,000 | 0,014 | |
| Chloroflexi; Thermomicrobia | 0,000 | | 0,002 | 0,000 | 0,024 | 0,000 | | 0,000 | | 0,000 | 0,000 | 0,003 | |
| Cyanobacteria; Other | 0,003 | | 0,016 | 0,000 | 0,028 | 0,023 | | 0,000 | | 0,000 | 0,030 | 0,054 | |
| Cyanobacteria | 0,000 | | 0,000 | 0,000 | 0,001 | 0,000 | | 0,000 | | 0,000 | 0,003 | 0,001 | |
| Cyanobacteria; 4C0d-2 | 0,000 | | 0,000 | 0,000 | 0,006 | 0,003 | | 0,003 | | 0,000 | 0,004 | 0,008 | |
| Cyanobacteria; Gloeobacterophycideae | 0,000 | | 0,001 | 0,000 | 0,000 | 0,000 | | 0,003 | | 0,000 | 0,000 | 0,000 | |
| Cyanobacteria; ML635J-21 | 0,001 | | 0,001 | 0,000 | 0,007 | 0,001 | | 0,014 | | 0,000 | 0,006 | 0,001 | |
| Cyanobacteria; Nostocophycideae | 0,000 | | 0,002 | 0,000 | 0,007 | 0,000 | | 0,000 | | 0,000 | 0,003 | 0,014 | |
| Cyanobacteria; Oscillatoriophycideae | 0,036 | | 0,065 | 0,000 | 0,364 | 0,006 | | 0,361 | | 0,001 | 1,817 | 1,106 | |
| Cyanobacteria; Synechococcophycideae | 0,022 | | 0,047 | 0,000 | 0,051 | 0,027 | | 0,139 | | 0,002 | 0,077 | 0,814 | |
| Deferribacteres; Deferribacteres | 0,000 | | 0,002 | 0,000 | 0,000 | 0,000 | | 0,001 | | 0,000 | 0,000 | 0,000 | |
| Elusimicrobia; Other | 0,004 | | 0,007 | 0,000 | 0,001 | 0,001 | | 0,000 | | 0,000 | 0,000 | 0,004 | |
| Elusimicrobia | 0,000 | | 0,002 | 0,000 | 0,001 | 0,000 | | 0,000 | | 0,000 | 0,000 | 0,000 | |
| Elusimicrobia; Elusimicrobia | 0,057 | | 0,062 | 0,000 | 0,032 | 0,001 | | 0,011 | | 0,000 | 0,002 | 0,009 | |
| Elusimicrobia; Endomicrobia | 0,000 | | 0,003 | 0,000 | 0,001 | 0,001 | | 0,000 | | 0,000 | 0,000 | 0,000 | |
| Elusimicrobia; OP2 | 0,003 | | 0,002 | 0,000 | 0,000 | 0,001 | | 0,000 | | 0,000 | 0,000 | 0,000 | |
| FCPU426 | 0,003 | | 0,001 | 0,000 | 0,000 | 0,000 | | 0,002 | | 0,000 | 0,001 | 0,004 | |
| Fibrobacteres; Other | 0,002 | | 0,000 | 0,000 | 0,001 | 0,000 | | 0,001 | | 0,000 | 0,000 | 0,001 | |
| Fibrobacteres | 0,003 | | 0,004 | 0,000 | 0,000 | 0,000 | | 0,002 | | 0,000 | 0,001 | 0,000 | |
| Fibrobacteres; B5-096 | 0,003 | | 0,007 | 0,000 | 0,008 | 0,001 | | 0,000 | | 0,000 | 0,003 | 0,004 | |
| Fibrobacteres; Fibrobacteria | 0,137 | | 0,203 | 0,004 | 0,068 | 0,022 | | 0,028 | | 0,002 | 0,026 | 0,051 | |
| Fibrobacteres; TG3 | 0,114 | | 0,155 | 0,001 | 0,013 | 0,034 | | 0,017 | | 0,000 | 0,013 | 0,018 | |
| Firmicutes; Other | 0,001 | | 0,002 | 0,000 | 0,001 | 0,004 | | 0,010 | | 0,001 | 0,002 | 0,023 | |
| Firmicutes; Bacilli | 0,013 | | 0,015 | 0,000 | 0,013 | 0,001 | | 0,003 | | 0,000 | 0,004 | 0,006 | |
| Firmicutes; Clostridia | 0,566 | | 0,518 | 0,007 | 0,543 | 0,211 | | 6,698 | | 0,090 | 2,717 | 2,620 | |
| Fusobacteria; Fusobacteriia | 0,046 | | 0,043 | 0,000 | 0,065 | 0,035 | | 0,044 | | 0,001 | 0,503 | 2,732 | |
| GN02; Other | 0,000 | | 0,000 | 0,000 | 0,000 | 0,000 | | 0,001 | | 0,000 | 0,001 | 0,000 | |
| GN02 | 0,003 | | 0,004 | 0,000 | 0,006 | 0,017 | | 0,074 | | 0,001 | 0,016 | 0,020 | |
| GN02; 3BR-5F | 0,022 | | 0,019 | 0,000 | 0,023 | 0,004 | | 0,035 | | 0,001 | 0,015 | 0,062 | |
| GN02; BB34 | 0,019 | | 0,035 | 0,000 | 0,044 | 0,011 | | 0,042 | | 0,001 | 0,014 | 0,082 | |
| GN02; BD1-5 | 0,002 | | 0,007 | 0,000 | 0,005 | 0,047 | | 0,050 | | 0,000 | 0,051 | 0,064 | |
| GN02; GKS2-174 | 0,006 | | 0,003 | 0,000 | 0,003 | 0,000 | | 0,022 | | 0,000 | 0,022 | 0,011 | |
| GN02; GN07 | 0,004 | | 0,010 | 0,000 | 0,003 | 0,000 | | 0,001 | | 0,000 | 0,001 | 0,015 | |
| GN02; GN10 | 0,001 | | 0,004 | 0,000 | 0,000 | 0,000 | | 0,002 | | 0,000 | 0,001 | 0,004 | |
| GN02; IIB17 | 0,005 | | 0,001 | 0,000 | 0,005 | 0,002 | | 0,011 | | 0,000 | 0,003 | 0,012 | |
| GN04; Other | 0,005 | | 0,006 | 0,000 | 0,008 | 0,000 | | 0,002 | | 0,000 | 0,000 | 0,001 | |
| GN04 | 0,024 | | 0,042 | 0,001 | 0,026 | 0,000 | | 0,009 | | 0,001 | 0,002 | 0,070 | |
| GN04; 5bav_B12 | 0,004 | | 0,003 | 0,000 | 0,007 | 0,000 | | 0,001 | | 0,000 | 0,000 | 0,007 | |
| GN04; GN15 | 0,061 | | 0,048 | 0,001 | 0,039 | 0,001 | | 0,015 | | 0,001 | 0,005 | 0,066 | |
| GN04; MSB-5A5 | 0,000 | | 0,001 | 0,000 | 0,000 | 0,000 | | 0,000 | | 0,000 | 0,000 | 0,000 | |
| GOUTA4 | 0,000 | | 0,000 | 0,000 | 0,002 | 0,000 | | 0,000 | | 0,000 | 0,000 | 0,005 | |
| Gemmatimonadetes; Other | 0,001 | | 0,001 | 0,000 | 0,001 | 0,000 | | 0,001 | | 0,000 | 0,001 | 0,002 | |
| Gemmatimonadetes | 0,000 | | 0,000 | 0,000 | 0,063 | 0,000 | | 0,000 | | 0,000 | 0,000 | 0,000 | |
| Gemmatimonadetes; Gemm-1 | 0,106 | | 0,126 | 0,002 | 0,155 | 0,006 | | 0,036 | | 0,001 | 0,013 | 0,109 | |
| Gemmatimonadetes; Gemm-2 | 0,290 | | 0,392 | 0,004 | 0,929 | 0,033 | | 0,323 | | 0,006 | 0,213 | 0,630 | |
| Gemmatimonadetes; Gemm-4 | 0,466 | | 0,527 | 0,004 | 0,347 | 0,014 | | 0,072 | | 0,001 | 0,089 | 0,043 | |
| Gemmatimonadetes; Gemm-5 | 0,011 | | 0,003 | 0,000 | 0,011 | 0,001 | | 0,000 | | 0,000 | 0,001 | 0,007 | |
| Gemmatimonadetes; Gemm-6 | 0,000 | | 0,000 | 0,000 | 0,000 | 0,000 | | 0,000 | | 0,000 | 0,000 | 0,001 | |
| Gemmatimonadetes; JL-ETNP-Z39 | 0,004 | | 0,012 | 0,000 | 0,015 | 0,001 | | 0,001 | | 0,000 | 0,000 | 0,028 | |
| H-178 | 0,016 | | 0,023 | 0,000 | 0,001 | 0,009 | | 0,022 | | 0,001 | 0,007 | 0,034 | |
| Hyd24-12; WM88 | 0,016 | | 0,009 | 0,000 | 0,000 | 0,002 | | 0,028 | | 0,001 | 0,005 | 0,021 | |
| KSB3; Other | 0,015 | | 0,008 | 0,000 | 0,001 | 0,001 | | 0,001 | | 0,000 | 0,003 | 0,007 | |
| KSB3 | 0,099 | | 0,112 | 0,002 | 0,017 | 0,009 | | 0,032 | | 0,001 | 0,029 | 0,079 | |
| KSB3; GN06 | 0,000 | | 0,000 | 0,000 | 0,000 | 0,000 | | 0,000 | | 0,000 | 0,000 | 0,000 | |
| Kazan-3B-28 | 0,000 | | 0,000 | 0,000 | 0,003 | 0,000 | | 0,000 | | 0,000 | 0,000 | 0,001 | |
| LCP-89; SAW1_B44 | 0,053 | | 0,069 | 0,001 | 0,002 | 0,002 | | 0,027 | | 0,001 | 0,002 | 0,047 | |
| LD1 | 0,006 | | 0,002 | 0,000 | 0,001 | 0,000 | | 0,000 | | 0,000 | 0,000 | 0,010 | |
| Lentisphaerae; [Lentisphaeria] | 0,191 | | 0,388 | 0,004 | 0,402 | 0,075 | | 0,096 | | 0,001 | 0,086 | 0,225 | |
| MVS-104 | 0,002 | | 0,007 | 0,000 | 0,000 | 0,000 | | 0,000 | | 0,000 | 0,000 | 0,004 | |
| NKB19; Other | 0,000 | | 0,003 | 0,000 | 0,006 | 0,000 | | 0,002 | | 0,000 | 0,003 | 0,002 | |
| NKB19 | 0,009 | | 0,017 | 0,000 | 0,047 | 0,001 | | 0,009 | | 0,000 | 0,003 | 0,029 | |
| NKB19; SHAB590 | 0,000 | | 0,000 | 0,000 | 0,013 | 0,000 | | 0,000 | | 0,000 | 0,005 | 0,001 | |
| NKB19; TSBW08 | 0,005 | | 0,003 | 0,000 | 0,033 | 0,001 | | 0,025 | | 0,000 | 0,011 | 0,010 | |
| Nitrospirae; Nitrospira | 0,049 | | 0,076 | 0,001 | 0,810 | 0,001 | | 0,004 | | 0,000 | 0,010 | 0,145 | |
| OD1; Other | 0,005 | | 0,006 | 0,000 | 0,008 | 0,005 | | 0,011 | | 0,000 | 0,001 | 0,007 | |
| OD1 | 0,005 | | 0,003 | 0,000 | 0,007 | 0,010 | | 0,067 | | 0,002 | 0,024 | 0,091 | |
| OD1; ABY1 | 0,028 | | 0,060 | 0,001 | 0,112 | 0,027 | | 0,234 | | 0,003 | 0,051 | 0,140 | |
| OD1; Mb-NB09 | 0,000 | | 0,005 | 0,000 | 0,042 | 0,001 | | 0,007 | | 0,000 | 0,001 | 0,027 | |
| OD1; SM2F11 | 0,000 | | 0,000 | 0,000 | 0,003 | 0,000 | | 0,000 | | 0,000 | 0,000 | 0,001 | |
| OD1; ZB2 | 0,072 | | 0,073 | 0,001 | 0,130 | 0,145 | | 0,196 | | 0,002 | 0,249 | 0,197 | |
| OP1; MSBL6 | 0,006 | | 0,002 | 0,000 | 0,000 | 0,000 | | 0,001 | | 0,000 | 0,001 | 0,004 | |
| OP1; [Acetothermia] | 0,002 | | 0,000 | 0,000 | 0,014 | 0,000 | | 0,000 | | 0,000 | 0,000 | 0,004 | |
| OP11; OP11-3 | 0,000 | | 0,000 | 0,000 | 0,001 | 0,000 | | 0,000 | | 0,000 | 0,000 | 0,000 | |
| OP3; Other | 0,006 | | 0,002 | 0,000 | 0,004 | 0,001 | | 0,003 | | 0,000 | 0,001 | 0,005 | |
| OP3 | 0,004 | | 0,009 | 0,000 | 0,001 | 0,002 | | 0,000 | | 0,000 | 0,000 | 0,002 | |
| OP3; BD4-9 | 0,045 | | 0,032 | 0,001 | 0,029 | 0,014 | | 0,002 | | 0,000 | 0,001 | 0,022 | |
| OP3; PBS-25 | 0,031 | | 0,047 | 0,000 | 0,102 | 0,008 | | 0,008 | | 0,000 | 0,012 | 0,017 | |
| OP3; koll11 | 0,026 | | 0,031 | 0,000 | 0,024 | 0,007 | | 0,001 | | 0,000 | 0,001 | 0,017 | |
| OP8; OP8_1 | 0,218 | | 0,157 | 0,003 | 0,001 | 0,009 | | 0,012 | | 0,001 | 0,002 | 0,108 | |
| OP8; OP8_2 | 0,107 | | 0,109 | 0,002 | 0,007 | 0,003 | | 0,054 | | 0,001 | 0,021 | 0,113 | |
| OP8; SAW1_B6 | 0,003 | | 0,003 | 0,000 | 0,000 | 0,000 | | 0,002 | | 0,000 | 0,000 | 0,009 | |
| OP9; JS1 | 0,000 | | 0,000 | 0,000 | 0,000 | 0,000 | | 0,000 | | 0,000 | 0,000 | 0,000 | |
| PAUC34f; | 0,002 | | 0,002 | 0,000 | 0,003 | 0,000 | | 0,000 | | 0,000 | 0,001 | 0,004 | |
| Planctomycetes; Other | 0,068 | | 0,096 | 0,001 | 0,085 | 0,006 | | 0,024 | | 0,000 | 0,021 | 0,027 | |
| Planctomycetes; | 0,138 | | 0,177 | 0,002 | 0,217 | 0,004 | | 0,029 | | 0,000 | 0,034 | 0,104 | |
| Planctomycetes; 028H05-P-BN-P5 | 0,008 | | 0,004 | 0,000 | 0,032 | 0,001 | | 0,033 | | 0,000 | 0,053 | 0,029 | |
| Planctomycetes; BD7-11 | 0,014 | | 0,016 | 0,000 | 0,060 | 0,001 | | 0,002 | | 0,000 | 0,001 | 0,020 | |
| Planctomycetes; C6 | 0,239 | | 0,323 | 0,004 | 0,262 | 0,012 | | 0,160 | | 0,003 | 0,180 | 0,275 | |
| Planctomycetes; ODP123 | 0,005 | | 0,008 | 0,000 | 0,009 | 0,001 | | 0,003 | | 0,000 | 0,005 | 0,012 | |
| Planctomycetes; OM190 | 0,178 | | 0,334 | 0,002 | 0,640 | 0,026 | | 0,264 | | 0,002 | 0,323 | 0,372 | |
| Planctomycetes; Phycisphaerae | 0,363 | | 0,445 | 0,008 | 1,356 | 0,019 | | 0,276 | | 0,005 | 0,370 | 0,644 | |
| Planctomycetes; Pla3 | 0,144 | | 0,165 | 0,002 | 0,150 | 0,001 | | 0,010 | | 0,000 | 0,008 | 0,038 | |
| Planctomycetes; Pla4 | 0,002 | | 0,009 | 0,000 | 0,001 | 0,000 | | 0,000 | | 0,000 | 0,000 | 0,002 | |
| Planctomycetes; Planctomycetia | 1,099 | | 1,398 | 0,014 | 1,656 | 0,156 | | 0,965 | | 0,010 | 1,313 | 1,027 | |
| Planctomycetes; [Brocadiae] | 0,000 | | 0,000 | 0,000 | 0,012 | 0,000 | | 0,000 | | 0,000 | 0,000 | 0,001 | |
| Planctomycetes; vadinHA49 | 0,030 | | 0,048 | 0,000 | 0,102 | 0,008 | | 0,028 | | 0,000 | 0,009 | 0,021 | |
| Poribacteria; | 0,000 | | 0,000 | 0,000 | 0,002 | 0,000 | | 0,000 | | 0,000 | 0,000 | 0,000 | |
| Proteobacteria; Other | 0,552 | | 0,466 | 0,004 | 0,152 | 0,261 | | 0,261 | | 0,001 | 0,192 | 0,143 | |
| Proteobacteria; Alphaproteobacteria | 1,577 | | 2,743 | 0,017 | 14,237 | 28,726 | | 8,796 | | 0,079 | 15,270 | 13,954 | |
| Proteobacteria; Betaproteobacteria | 0,004 | | 0,012 | 0,000 | 0,207 | 0,292 | | 0,078 | | 0,001 | 0,036 | 0,044 | |
| Proteobacteria; Deltaproteobacteria | 25,374 | | 23,311 | 0,258 | 10,357 | 1,266 | | 9,789 | | 0,128 | 6,805 | 13,568 | |
| Proteobacteria; Epsilonproteobacteria | 2,748 | | 3,820 | 0,044 | 0,267 | 0,828 | | 13,889 | | 0,043 | 2,134 | 2,114 | |
| Proteobacteria; Gammaproteobacteria | 38,043 | | 35,465 | 0,322 | 40,895 | 37,738 | | 13,759 | | 0,157 | 24,864 | 23,705 | |
| Proteobacteria; TA18 | 0,023 | | 0,027 | 0,000 | 0,166 | 0,013 | | 0,068 | | 0,001 | 0,186 | 0,116 | |
| Proteobacteria; Zetaproteobacteria | 0,000 | | 0,006 | 0,000 | 0,003 | 0,003 | | 0,000 | | 0,000 | 0,000 | 0,003 | |
| SAR406; AB16 | 0,041 | | 0,072 | 0,001 | 0,057 | 0,011 | | 0,064 | | 0,002 | 0,019 | 0,070 | |
| SBR1093; | 0,000 | | 0,000 | 0,000 | 0,015 | 0,000 | | 0,000 | | 0,000 | 0,000 | 0,001 | |
| SBR1093; EC214 | 0,000 | | 0,000 | 0,000 | 0,066 | 0,000 | | 0,000 | | 0,000 | 0,000 | 0,000 | |
| SBR1093; VHS-B5-50 | 0,000 | | 0,002 | 0,000 | 0,095 | 0,000 | | 0,002 | | 0,000 | 0,006 | 0,022 | |
| SR1; | 0,002 | | 0,004 | 0,000 | 0,002 | 0,004 | | 0,062 | | 0,001 | 0,046 | 0,021 | |
| Spirochaetes; Other | 0,005 | | 0,007 | 0,000 | 0,000 | 0,000 | | 0,001 | | 0,000 | 0,000 | 0,001 | |
| Spirochaetes; GN05 | 0,005 | | 0,006 | 0,000 | 0,000 | 0,000 | | 0,008 | | 0,000 | 0,004 | 0,008 | |
| Spirochaetes; MVP-15 | 0,002 | | 0,001 | 0,000 | 0,001 | 0,001 | | 0,002 | | 0,000 | 0,002 | 0,010 | |
| Spirochaetes; Spirochaetes | 0,940 | | 1,115 | 0,016 | 0,394 | 0,144 | | 0,752 | | 0,009 | 0,277 | 0,602 | |
| Spirochaetes; [Brachyspirae] | 0,030 | | 0,032 | 0,001 | 0,003 | 0,002 | | 0,001 | | 0,000 | 0,002 | 0,020 | |
| Spirochaetes; [Brevinematae] | 0,008 | | 0,004 | 0,000 | 0,002 | 0,001 | | 0,002 | | 0,000 | 0,008 | 0,001 | |
| Spirochaetes; [Leptospirae] | 0,034 | | 0,035 | 0,000 | 0,005 | 0,003 | | 0,027 | | 0,001 | 0,019 | 0,069 | |
| TM6; Other | 0,000 | | 0,001 | 0,000 | 0,004 | 0,000 | | 0,002 | | 0,000 | 0,001 | 0,002 | |
| TM6; | 0,000 | | 0,001 | 0,000 | 0,001 | 0,001 | | 0,000 | | 0,000 | 0,002 | 0,000 | |
| TM6; SBRH58 | 0,009 | | 0,013 | 0,000 | 0,020 | 0,005 | | 0,025 | | 0,001 | 0,009 | 0,052 | |
| TM6; SJA-4 | 0,009 | | 0,009 | 0,000 | 0,055 | 0,003 | | 0,006 | | 0,001 | 0,009 | 0,021 | |
| TM7; Other | 0,000 | | 0,000 | 0,000 | 0,002 | 0,001 | | 0,010 | | 0,000 | 0,036 | 0,022 | |
| TM7; | 0,000 | | 0,003 | 0,000 | 0,012 | 0,010 | | 0,065 | | 0,000 | 0,062 | 0,050 | |
| TM7; TM7-1 | 0,013 | | 0,008 | 0,000 | 0,025 | 0,017 | | 0,041 | | 0,001 | 0,136 | 0,046 | |
| TM7; TM7-3 | 0,000 | 0,000 | | 0,000 | 0,000 | 0,001 | 0,000 | | 0,000 | | 0,000 | 0,000 |  |
| TPD-58; | 0,000 | | 0,001 | 0,000 | 0,001 | 0,000 | | 0,001 | | 0,000 | 0,000 | 0,002 | |
| Tenericutes; Other | 0,002 | | 0,004 | 0,000 | 0,000 | 0,000 | | 0,001 | | 0,000 | 0,003 | 0,000 | |
| Tenericutes; | 0,000 | | 0,000 | 0,000 | 0,000 | 0,000 | | 0,001 | | 0,000 | 0,000 | 0,000 | |
| Tenericutes; Mollicutes | 0,011 | | 0,037 | 0,000 | 0,042 | 0,043 | | 0,038 | | 0,000 | 0,020 | 0,033 | |
| Verrucomicrobia; Other | 0,013 | | 0,019 | 0,000 | 0,016 | 0,003 | | 0,208 | | 0,000 | 0,121 | 0,038 | |
| Verrucomicrobia; | 0,000 | | 0,000 | 0,000 | 0,000 | 0,000 | | 0,002 | | 0,000 | 0,000 | 0,001 | |
| Verrucomicrobia; Opitutae | 0,068 | | 0,078 | 0,000 | 0,240 | 0,029 | | 0,119 | | 0,001 | 0,061 | 0,067 | |
| Verrucomicrobia; Verruco-5 | 0,602 | | 0,733 | 0,009 | 0,958 | 0,083 | | 1,797 | | 0,018 | 0,635 | 1,086 | |
| Verrucomicrobia; Verrucomicrobiae | 0,175 | | 0,453 | 0,002 | 1,414 | 0,099 | | 2,211 | | 0,018 | 2,485 | 1,348 | |
| Verrucomicrobia; [Methylacidiphilae] | 0,000 | | 0,000 | 0,000 | 0,015 | 0,000 | | 0,000 | | 0,000 | 0,002 | 0,012 | |
| Verrucomicrobia; [Pedosphaerae] | 0,466 | | 0,654 | 0,004 | 0,390 | 0,009 | | 0,032 | | 0,000 | 0,016 | 0,030 | |
| Verrucomicrobia; [Spartobacteria] | 0,005 | | 0,009 | 0,000 | 0,000 | 0,000 | | 0,044 | | 0,000 | 0,023 | 0,010 | |
| WPS-2; | 0,000 | | 0,002 | 0,000 | 0,000 | 0,002 | | 0,005 | | 0,000 | 0,029 | 0,052 | |
| WS2; Other | 0,000 | | 0,000 | 0,000 | 0,000 | 0,000 | | 0,002 | | 0,000 | 0,001 | 0,003 | |
| WS2; | 0,002 | | 0,003 | 0,000 | 0,000 | 0,000 | | 0,000 | | 0,000 | 0,000 | 0,000 | |
| WS2; Kazan-3B-09 | 0,006 | | 0,003 | 0,000 | 0,005 | 0,000 | | 0,017 | | 0,000 | 0,003 | 0,015 | |
| WS2; SHA-109 | 0,016 | | 0,012 | 0,000 | 0,047 | 0,001 | | 0,033 | | 0,001 | 0,021 | 0,065 | |
| WS3; PRR-12 | 1,420 | | 1,653 | 0,018 | 0,841 | 0,050 | | 1,108 | | 0,022 | 0,514 | 1,444 | |
| WS5; | 0,001 | | 0,000 | 0,000 | 0,000 | 0,001 | | 0,000 | | 0,000 | 0,000 | 0,000 | |
| WWE1; [Cloacamonae] | 0,011 | | 0,022 | 0,000 | 0,000 | 0,007 | | 0,216 | | 0,004 | 0,012 | 0,087 | |
| ZB3; Other | 0,000 | | 0,000 | 0,000 | 0,003 | 0,000 | | 0,000 | | 0,000 | 0,000 | 0,001 | |
| ZB3; | 0,004 | | 0,002 | 0,000 | 0,004 | 0,001 | | 0,000 | | 0,000 | 0,000 | 0,010 | |
| ZB3; BS119 | 0,001 | | 0,004 | 0,000 | 0,000 | 0,002 | | 0,001 | | 0,000 | 0,000 | 0,003 | |
| [Caldithrix]; KSB1 | 0,042 | | 0,038 | 0,001 | 0,038 | 0,004 | | 0,006 | | 0,000 | 0,002 | 0,019 | |
| [Thermi]; Deinococci | 0,000 | | 0,001 | 0,000 | 0,035 | 0,000 | | 0,020 | | 0,000 | 0,045 | 0,044 | |
