# Supplementary material for: Rhizosphere Microbiomes of European Seagrasses Are Selected by the Plant, But Are Not Species Specific
Source: Front Microbiol. 2016 Mar 31;7:440. doi: 10.3389/fmicb.2016.00440 (PMC4815253; doi:10.3389/fmicb.2016.00440)
Supplement: Supplementary file 3 [file Table_3.DOCX]

Supplementary Table S3

Rhizosphere microbiomes of European seagrasses are selected by the plant, but are not species specific

Catarina Cúcio, Aschwin H. Engelen, Rodrigo Costa, Gerard Muyzer*

*** Correspondence:** g.muijzer@uva.nl

Supplementary Table S3 – Core rhizobiome of seagrasses. Classification of the 101 OTUs common to the seagrasses *Z. marina*, *Z. noltii* and *C. nodosa* from Portugal and France. The number of sequences indicates the absolute number of reads sequenced from seagrass samples.

| Phylum | | Class | Order | Family | | Genus | # Sequences | # OTUs |
| --- | --- | --- | --- | --- | --- | --- | --- | --- |
| *Acidobacteria* | |  |  |  | |  | **1879** | **5** |
|  | | Acidobacteria-6 | CCU21 | - | | - | 151 | 1 |
|  | | OS-K | - | - | | - | 1572 | 3 |
|  | | RB25 | - | - | | - | 156 | 1 |
| *Actinobacteria* | |  |  |  | |  | **10020** | **7** |
|  | | *Acidimicrobiia* | *Acidimicrobiales* | Acidimicrobiaceae | | Illumatobacter* | 621 | 1 |
|  | |  |  | C111 | | - | 547 | 1 |
|  | |  |  | Koll13 | | - | 8852 | 5 |
| *Bacteroidetes* | |  |  |  | |  | **17614** | **21** |
|  | | *Bacteroidia* | *Bacteroidales* | - | | - | 6461 | 8 |
|  | |  |  | SB-1 | | - | 2373 | 2 |
|  | | *Cytophagia* | *Cytophagales* | *Flammeovirgaceae* | | - | 822 | 1 |
|  | | *Flavobacteriia* | *Flavobacteriales* | *Flavobacteriaceae* | | *Lutimonas* | 5283 | 3 |
|  | |  |  |  | | *Robiginitalea* | 604 | 1 |
|  | |  |  |  | | - | 1203 | 2 |
|  | | - | - | - | | - | 868 | 4 |
| *Caldithrix* | |  |  |  | |  | **670** | **1** |
|  | | *Caldithrixae* | *Caldithrixales* | *Caldithrixaceae* | | LCP-26 | 670 | 1 |
| *Chloroflexi* | |  |  |  | |  | **434** | **2** |
|  | | *Anaerolineae* | S0208 | - | | - | 318 | 1 |
|  | | Ellin6529 | - | - | | - | 116 | 1 |
| *Firmicutes* | |  |  |  | |  | **8436** | **2** |
|  | | *Clostridia* | *Clostridiales* | - | | - | 8436 | 2 |
| *Gemmatimonadetes* | |  |  |  | |  | **544** | **1** |
|  | | Gemm-4 | - | - | | - | 544 | 1 |
| *Proteobacteria* | |  |  |  | |  | **73130** | **59** |
|  | | *Alphaproteobacteria* |  |  | |  | 3172 | 7 |
|  | |  | *Rhizobiales* | *Hyphomicrobiaceae* | | *Hyphomicrobium* | 101 | 1 |
|  | |  |  |  | | - | 742 | 2 |
|  | |  | *Rhizobiales* | *Phyllobacteriaceae* | | - | 215 | 1 |
|  | |  | *Rhodobacterales* | *Rhodobacteraceae* | | *Phaeobacter* | 1638 | 1 |
|  | |  | *Rhodobacterales* | *Rhodobacteraceae* | | - | 476 | 2 |
|  | | Deltaproteobacteria |  |  | |  | 22604 | 23 |
|  | |  | *Desulfarculales* | *Desulfarculaceae* | | - | 142 | 1 |
|  | |  | *Desulfobacterales* | *Desulfobulbaceae* | | *Desulfocapsa* | 223 | 1 |
|  | |  |  |  | | - | 5505 | 7 |
|  | |  |  | *Desulfobacteraceae* | | *Desulfococcus* | 8236 | 5 |
|  | |  |  |  | | *Desulfosarcina* | 1579 | 1 |
|  | |  |  |  | | - | 5399 | 5 |
|  | |  | *Myxococcales* | - | | - | 949 | 1 |
|  | |  | NB1-J | - | | - | 103 | 1 |
|  | |  | - | - | | - | 468 | 1 |
|  | | *Epsilonproteobacteria* |  |  | |  | 16649 | 3 |
|  | |  | *Campylobacterales* | *Helicobacteraceae* | | *Sulfurimonas* | 716 | 1 |
|  | |  |  |  | | - | 15933 | 2 |
|  | | *Gammaproteobacteria* |  |  | |  | 30705 | 26 |
|  | |  | *Alteromonadales* | OM60 | | - | 3216 | 7 |
|  | |  |  | - | | - | 116 | 1 |
| *Proteobacteria* (cont.) | | |  |  | |  |  |  |
|  | | *Gammaproteobacteria* (cont.) | |  | |  |  |  |
|  | |  | *Chromatiales* | - | | - | 12319 | 7 |
|  | |  | *Marinicellales* | *Marinicellaceae* | | - | 3776 | 5 |
|  | |  | *Thiohalorhabdales* | - | | - | 2844 | 1 |
|  | |  | *Thiotrichales* | *Piscirickettsiaceae* | | - | 1509 | 3 |
|  | |  |  | *Thiotrichaceae* | | - | 2560 | 1 |
|  | |  | - | - | | - | 4365 | 1 |
| WS3 | |  |  |  | |  | **1181** | **3** |
|  | | PRR-12 | GN03 | KSB4 | | - | 1181 | 3 |
|  | |  |  |  | | **TOTAL** | **113908** | **101** |
| *- refers to unclassified OTUs.*  * Identification based on phylogenetic analysis using ARB | | | |  |  |  |  |  |
